# The soil organic matter decomposition mechanisms in ectomycorrhizal fungi are tuned for liberating soil organic nitrogen

**DOI:** 10.1038/s41396-018-0331-6

**Published:** 2018-12-11

**Authors:** César Nicolás, Tomas Martin-Bertelsen, Dimitrios Floudas, Johan Bentzer, Mark Smits, Tomas Johansson, Carl Troein, Per Persson, Anders Tunlid

**Affiliations:** 1 Department of Biology, Microbial Ecology Group, Lund University, Ecology Building, SE-223 62 Lund, Sweden; 2 Department of Astronomy and Theoretical Physics, Computational Biology and Biological Physics, Lund University, Sölvegatan 14A, SE-223 62 Lund, Sweden; 3 Centre for Environmental Sciences, Hasselt University, Building D, Agoralaan, 3590 Diepenbeck, Limburg, Belgium; 4 Centre for Environmental and Climate Research (CEC), Lund University, Ecology Building, SE-223 62 Lund, Sweden

**Keywords:** Microbial ecology, Biogeochemistry, Fungi

## Abstract

Many trees form ectomycorrhizal symbiosis with fungi. During symbiosis, the tree roots supply sugar to the fungi in exchange for nitrogen, and this process is critical for the nitrogen and carbon cycles in forest ecosystems. However, the extents to which ectomycorrhizal fungi can liberate nitrogen and modify the soil organic matter and the mechanisms by which they do so remain unclear since they have lost many enzymes for litter decomposition that were present in their free-living, saprotrophic ancestors. Using time-series spectroscopy and transcriptomics, we examined the ability of two ectomycorrhizal fungi from two independently evolved ectomycorrhizal lineages to mobilize soil organic nitrogen. Both species oxidized the organic matter and accessed the organic nitrogen. The expression of those events was controlled by the availability of glucose and inorganic nitrogen. Despite those similarities, the decomposition mechanisms, including the type of genes involved as well as the patterns of their expression, differed markedly between the two species. Our results suggest that in agreement with their diverse evolutionary origins, ectomycorrhizal fungi use different decomposition mechanisms to access organic nitrogen entrapped in soil organic matter. The timing and magnitude of the expression of the decomposition activity can be controlled by the below-ground nitrogen quality and the above-ground carbon supply.

## Introduction

A large portion of nitrogen (N) in forest soils is found in organic form, primarily as amides and amines, but also as heterocyclic-N molecules [1]. These N molecules are associated with polyphenols, polysaccharides, lignin residues, lipids, and other degradation products of plant and microbial origin that are present in the soil organic matter (SOM) [2]. The capacity of forest trees to assimilate organic N is limited [3]. Access to organic N sources, such as proteins or chitin, requires decomposition to make the organic N molecules accessible and, subsequently, to liberate N from those molecules. Plants are generally thought to depend on microbial decomposition to access the soil N [4]. A long-standing hypothesis proposes that the ectomycorrhizal (ECM) fungal symbionts have a key role in this process [5, 6]; however, the extent of the involvement of ECM fungi in SOM decomposition and mobilization of N compounds is debated [7, 8].

ECM fungi evolved several times from saprotrophic ancestors [9, 10]. These ancestors probably utilized diverse decomposition strategies resembling those seen in white-rot (WR) wood decayers, which use an enzymatic system for the decomposition of lignocellulose; brown-rot (BR) wood decayers, which utilize a two-step mechanism involving hydroxyl radicals (∙OH) generated by Fenton chemistry and hydrolytic enzymes [11]; and litter decomposers, which presumably use enzymatic decomposition systems similar to those of WR fungi [12]. During the transition from saprotrophic to symbiotic lifestyle, ECM fungi lost a large number of plant cell wall-degrading enzymes (PCWDEs) [9, 10]. The convergent gene losses in relation to PCWDEs seen in ECM lineages have been used as an argument against a major role of ECM fungi in SOM decomposition [8]. However, ECM lineages have lost many, but not all, genes coding for PCWDEs, with diverse types and numbers of genes related to decomposition retained across lineages [10]. The high variability of the retained PCWDE-coding genes and the diverse evolutionary backgrounds of ECM lineages suggest that ECM fungi could have also retained and adapted some features of the decomposition mechanisms to the symbiotic lifestyle [13].

To what extent ECM fungi make use of the remaining decomposition systems is not well understood. At least some ECM fungi oxidize organic matter in a SOM extract in the presence of an energy source (i.e., glucose) [13, 14]. Furthermore, ECM *Cortinarius* species encode ligninolytic class-II peroxidases, whose gene transcription correlates with the peroxidase activity in the boreal forest soil, supporting the hypothesis that these species may play an important role in SOM decomposition [15]. In addition, decomposition activities of ECM fungi have been inferred by ecological studies that relied on enzymatic assays detecting the activity of various hydrolytic and oxidative PCWDEs [16, 17]. However, one limitation of such studies is that the assays are performed with ECM root tips and not the mycelium colonizing the soil substrate. Several of those assays, in particular the ones probing for oxidative enzyme activity, are unspecific and do not properly capture the decomposition activity [18]. Alternative hypotheses for the role of the detected enzymes include the decomposition of dead root tips [18] and remodeling of the root cell wall during host colonization [9, 19, 20].

Additionally, the environmental cues that regulate SOM decomposition in ECM fungi are not known. Laboratory experiments revealed that the oxidative decomposition system in the ECM fungus *Paxillus involutus* is expressed only in the presence of an energy source (i.e., glucose) [21]. In contrast, field studies based on enzyme assays suggest that ECM fungi can produce PCWDEs and metabolize SOM when the amount of carbon (C) supplied by the host plant is low [16]. Moreover, it is not known if the expression of the decomposition system of ECM fungi and the liberation of organic N compounds are concurrent. If so, the two processes might be regulated in conjunction and by similar nutritional signals, including the availability of inorganic and organic N sources.

To address these questions, we used time-series spectroscopy and transcriptomics to analyze two species of ECM fungi with independent evolutionary histories and contrasting growth characteristics. *P. involutus* is characterized by a rapidly growing mycelium, and a so-called long-distance exploration type [22]. The species is nested within a paraphyletic assemblage of BR wood decayers in the Boletales [10], and oxidizes SOM using a nonenzymatic Fenton-based system [14, 23]. By contrast, *Laccaria bicolor* develops a slow-growing, medium-distance smooth exploration subtype mycelium [22]. It belongs in the Agaricales and probably evolved from litter-decomposing saprotrophs [10]. The *L. bicolor* set of enzymes presumably involved in the degradation of PCW derived polymers is larger than that of *P. involutus*; several of these enzymes are expressed during growth on a SOM extract [10, 13].

## Materials and methods

### Fungal strains and culture conditions

Cultures of *P. involutus* (Batsch) Fr. (ATCC 200175) and *L. bicolor* (Maire) P.D. Orton (S238N) were grown on a layer of glass beads in Petri dishes containing a liquid minimum Melin-Norkrans (MMN) medium for 9 and 18 d, respectively (18 °C, in the dark) [14]. The medium was then replaced with MMN medium without N to induce the formation of an N-deprived mycelium. After 24 h, the mycelium was washed with sterile water and the SOM extract was added. The extract was supplemented with glucose to a final concentration similar to that in the MMN medium [14]. The soil was collected to a depth of 10 cm after removing recently fallen and partly decomposed litter from the top, in a 61-y-old Norway spruce stand growing in an N-poor site in central Sweden (soil pH, 5.0). It mainly consists of well-decomposed humus material with occasional occurrence of mineral soil. According to [24], mycelia of ECM fungi dominate therein.

SOM was extracted using hot water [25]. Further experimental details are given in the Supplementary Information.

### Chemical analyses

Total organic C, glucose, total N, and ammonium-N were determined as previously described [13]. Fourier-transform infrared (FTIR) spectra were recorded in diffuse reflectance mode on mixtures of freeze-dried samples and KBr (2% w/w) using a Vertex 80v spectrometer (Bruker Optics, Ettlingen, Germany). Each spectrum was the result of 128 consecutive scans at a resolution of 4 cm^−1^. KBr was used as background. The IR spectral data sets were analyzed by means of a multivariate curve resolution-alternating least squares method [26, 27]. Pyrolysis-gas chromatography/mass spectrometry was performed using a PerkinElmer Turbo-Mass/Autosystem XL with Frontier Lab Double-Shot Pyrolyser (PerkinElmer, Waltham, MA, USA). The ratio of 4-acetylguaiacol to *trans*-propenylguaiacol (Ox/C3-G) was used as a marker of the degree of side-chain degradation of lignin [28].

The Fe K-edge XANES spectra were collected at beamline I811, MaxLab, Lund, Sweden. The X-ray absorption spectra were processed and analyzed using the software SIXPack [29]. In order to obtain the redox state of Fe, the pre-edge peak was examined as described by Wilke et al. [30]. The centroid position of this peak is indicative of the Fe redox state. The integrated area and the centroid were calculated and one-way ANOVA was used for the statistical analysis of the centroids to establish differences between treatments.

The N K-edge XANES spectra (390–430 eV) were collected at the beamline 11ID-1 (SGM), Canadian Light Source, Saskatoon, Canada. The organic matter extract was poured on indium foil and dried at 30 °C. The main peaks of the XANES spectra were assigned by comparison with spectra of model compounds found in the literature [31]. In order to semiquantitatively compare the spectra, an ordination method (principal component analysis) based on the relative abundance of π* transitions (a–d) was used to find differences in the changes of organic N speciation between fungi. Further experimental details are provided in the Supplementary Information.

### Transcriptome analysis

Total RNA was isolated from the mycelium, and cDNA libraries were constructed as previously described [13]. The libraries were sequenced (RNA-Seq) using a HiSeq2000 instrument (Flow Cell v3) (Illumina Inc., San Diego, CA, USA) in single-read mode and with a read length of 50 bp (IGA Technology Services, Italy; www.igatechnology.com). Sequence reads were aligned to filtered gene models of *P. involutus* (ver. 1.0) [10] and *L. bicolor* (ver. 2.0) [9] using TopHat2 (ver. 2.0.13) [32]. Expression counts were determined using HTSeq (ver. 0.6.1) [33], and normalization factors were estimated using EDASeq (ver. 2.8.0) [34] and DESeq2 (ver. 1.14.1) [35]. Differentially expressed genes (DEGs) were identified using DESeq2, by testing for overall changes in expression during the time-course of the experiment using a likelihood ratio test, and for pairwise comparisons of (*t*_2_–*t*_4_) vs. *t*_1_ using a Wald test. *P* values were adjusted (*p*_adj_) for multiple testing using the Benjamini-Hochberg method [36] to control the false discovery rate with a significance cutoff of 0.01. KOG and KEGG enrichment analyses of DEGs were performed using the GOseq (ver. 1.26.0) tool [37].

The main temporal gene expression profiles were identified using the FunPat procedure [38] integrating annotation information, gene selection, and clustering. The input annotation information on genes encoding enzymes in metabolic pathways [39], organic N assimilation [40, 41], CAZymes, AAs, peroxidases [13], biosynthesis of secondary metabolites [13, 42] (Tables S1 and S2), autophagy [43], and secreted proteins (Fig. S1) were organized into a hierarchy to prioritize the identification of co-expressed genes associated with the most specific function. The average log_2_ gene expression for each time point was determined using DESeq2; only the annotated and highly changing time-DEGs were used as input in FunPat. The FunPat-clusters were classified into 15 identified response types (cf. Fig. 4). Statistical significance of the identified response types was evaluated by a permutation test of time points on the FunPat output.

To identify genes in *P. involutus* and *L. bicolor* potentially encoding proteins that are homologs to those being upregulated in *S. cerevisiae* during ammonium limitation, we collected a list of 41 proteins in *S. cerevisiae* that are significantly up-regulated during N-starvation conditions [44, 45]. The list was used as a query to identify putative homologs in the genomes of *P. involutus* and *L. bicolor* using BLASTP search (cut-off < 4.00E−20). One-to-one orthologous proteins were predicted using the reciprocal best alignment heuristic as implemented in Proteinortho (ver. 5) using the conserved synteny option [46].

A phylogenetic analysis of the yeast amino acid transporter (YAT) family was conducted by retrieving a preliminary dataset of all gene catalog proteins carrying the PF00324 domain from eight Agaricomycotina genomes [47]. A set of characterized sequences from *S. cerevisiae* and *Hebeloma cylindrosporum* [40] the YAT family was also included. A preliminary alignment was constructed using MAFFT [48] and poorly aligned regions were removed using Jalview [49]. A phylogenetic analysis was performed using RAxML [50] at Cipres [51] under the model PROTCATWAG, with 200 bootstrap runs. Further details are given in the Supplementary Information.

## Results

### Changes in the C and N content and the SOM composition associated with fungal activities

**Fig. 1:**
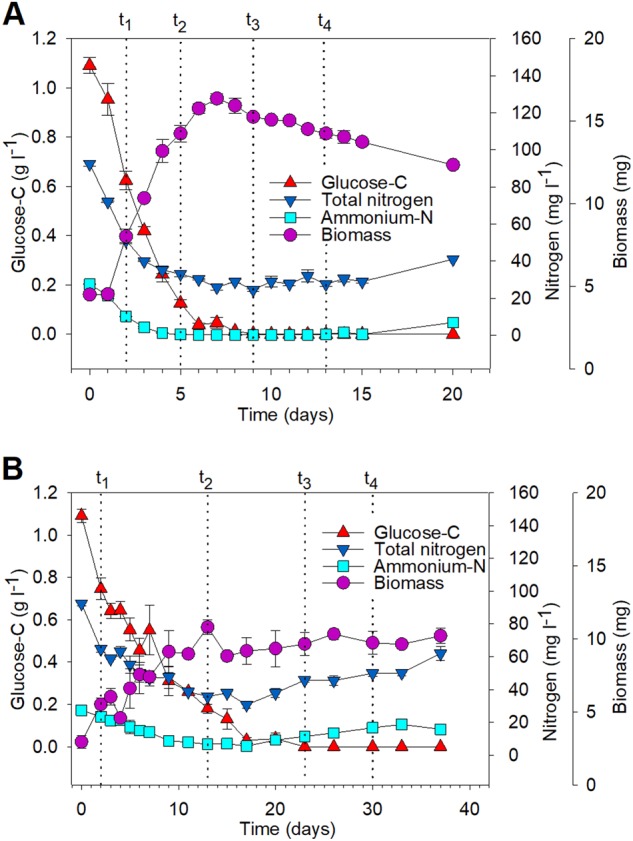
Mycelial biomass and chemical modifications of SOM extracts during decomposition by *P. involutus* (PAI) and *L. bicolor* (LAB). Dotted vertical lines indicate four time points (*t*_1_–*t*_4_) selected for spectroscopic and transcriptomic analyses. Changes in glucose-C, total N, ammonium-N, and the biomass of *P. involutus* (**A**) and *L. bicolor* (**B**) are shown (mean ± SE, *n* = 3). Note the different time-scales for the two fungi. Changes in the pH of the SOM extract are shown in Fig. S2B

*P. involutus* and *L. bicolor* were grown on SOM extracted by a hot-water method from the humic layer of a Norway spruce forest. At the start of the experiment, the extract was supplemented with glucose as an energy source. The total organic C and N content in the SOM extract decreased over time in the presence of both fungi (Fig. 1A, B, Fig. S2A). The N level in the SOM medium, in particular ammonium-N, dropped more rapidly than the C content in both cases. Nitrate was not detected in the SOM extract. The fungal biomass increased until glucose was depleted from the medium. At that moment, the biomass of *P. involutus* started to decline, while that of *L. bicolor* remained relatively constant (Fig. 1A, B).

Comparison of the FTIR spectra of the SOM extracts before and after incubation revealed a decrease of the bands associated with polysaccharides and ammonium, and an increase in the carbonyl band (C = O stretching) after the incubation (Fig. S3A, Table S3). The C = O stretching was more pronounced in the SOM extract incubated with *P. involutus* than the one incubated with *L. bicolor* (Fig. S3A). The infrared spectra of the SOM extract were resolved into four major components by the multivariate curve resolution-alternating least squares analysis, with a total explained variance equal to 99.99% (Fig. S3B). For both fungi, the first and second major resolved components decreased with time, which could be attributed to the uptake of nutrients (sugar-C and ammonium-N) from the medium. The third and fourth components increased with time, although they were not clearly resolved for *L. bicolor* (Fig. S3C).

Four time points were selected for a more detailed spectroscopy and transcriptome analysis (Fig. 1A, B). At *t*_1_, both glucose and ammonium were present in the SOM extract, and the fungal biomass increased. Thus, *t*_1_ represented an active growth (AG) phase. Next, *t*_2_ represented a phase when glucose was present, but ammonium was depleted from the medium (ND). At *t*_3_, glucose was not detected in the medium (<1 mg glucose–C l^−1^) and the mycelial growth was arrested, suggesting that the fungi were experiencing C depletion (CD). The last time point (*t*_4_) was chosen after the fungi would have experienced a prolonged C limitation (pCD).

### Lignin residue oxidation, iron speciation, and changes in organic N compound content

**Fig. 2:**
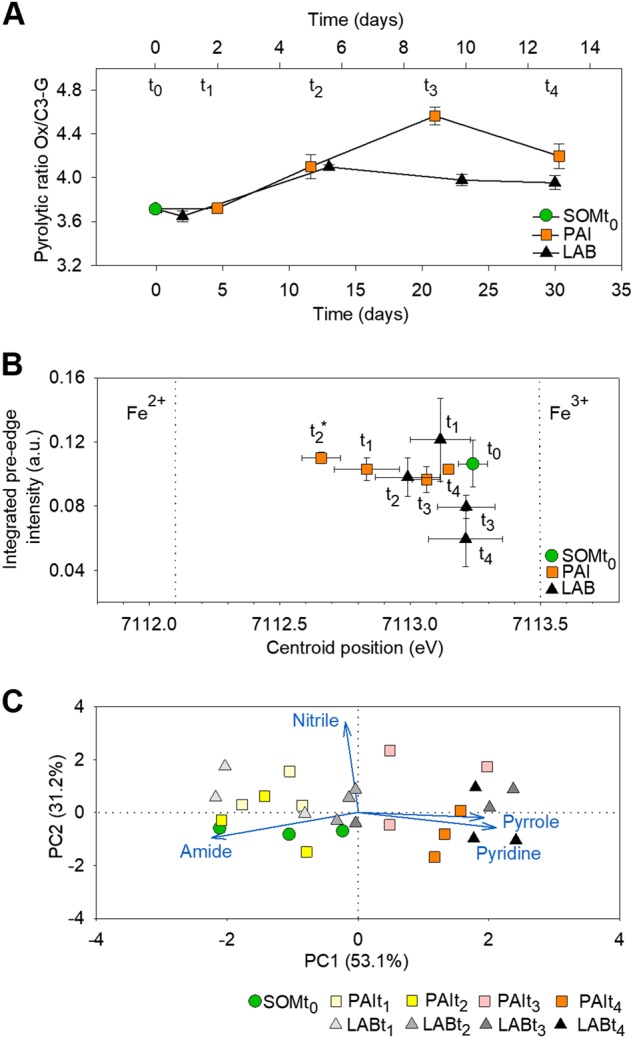
Lignin residue oxidation, iron speciation, and changes in organic N compound content. The time points *t*_1_–*t*_4_ are defined in Fig. 1. **A** Decomposition of lignin residues in the SOM extract, as determined by using pyrolysis-gas chromatography/mass spectrometry. The ratio of 4-acetylguaiacol to *trans*-propenylguaiacol (Ox/C3-G) is shown, as a marker of the degree of lignin side-chain degradation [13]. The data are corrected for the total organic C content and normalized to data for a SOM extract that had not been incubated with fungi (mean ± SE, *n* = 3). The top *x*-axis refers to the incubation time of *P. involutus* and the bottom *x*-axis refers to that of *L. bicolor*. **B** Changes in the speciation of iron in the SOM extract (mean ± SE, *n* = 2). Vertical lines indicate the centroid positions for ferric iron (Fe^3+^; at 7113.5 eV) or ferrous iron (Fe^2+^; at 7112.1 eV). The asterisk indicates significant difference from the initial SOM extract (SOMt_0_) (*p* < 0.05, one-way ANOVA). **c** The principal component analysis score and loading factor plot of the N K-edge spectra of the SOM extracts incubated with *P. involutus* and *L. bicolor*. The loading vectors were lengthened to improve the clarity of the figure

No oxidation of lignin residues was detected at the beginning of the SOM extract incubation with either fungus (AG, *t*_1_); in contrast, increasing oxidation was detected between *t*_1_ and *t*_2_ (ND) (Fig. 2A). The oxidation continued in the presence of *P. involutus*, but not *L. bicolor*, until *t*_3_. X-ray absorption spectroscopy at the Fe K-edge revealed iron reduction in the SOM extract, from ferric (Fe^3+^) to ferrous (Fe^2+^), during the AG and ND phases (*t*_1_ and *t*_2_) of *P. involutus*, and the iron was present in the oxidized form during the C starvation phases (*t*_3_ and *t*_4_) (Fig. 2B, Fig. S4). No significant changes in the iron oxidation state in the SOM extract were observed in the *L. bicolor* culture.

X-ray absorption spectroscopy at the N K-edge revealed that the relative abundance of various types of organic N compounds in the SOM extract (pyridine, nitrile, amide, and pyrrole) changed similarly during the incubation with *P. involutus* and *L. bicolor*, with a pronounced change between ND (*t*_2_) and CD (*t*_3_). Principal component analysis based on changes in relative abundances of these N compound types grouped SOM extracts at *t*_1_ and *t*_2_ together with the initial material, indicating low uptake of organic N by the fungi (Fig. 2C, Fig. S5, Table S4). As the incubation progressed to *t*_3_, SOM extracts incubated in the presence of *P. involutus* and *L. bicolor* were enriched in heterocyclic-N (pyridine and pyrrole) at the expense of amide, indicating uptake of amide compounds by the fungi (Fig. 2C). No separation of the samples based on the relative abundances of organic N types was observed in the PCA between *t*_3_ (CD) and *t*_4_ (pCD).

### Global transcriptional changes during SOM decomposition

The mycelial transcriptional profiles of the fungi were analyzed using RNA sequencing (Table S5). The transcriptomes of *P. involutus* and *L. bicolor* were distinct and different at the four evaluated time points (Fig. S6A). Overall, the expression of 43% (7779) of the predicted *P. involutus* genes and 39% (8919) of the predicted genes in *L. bicolor* was significantly altered over the time course of the experiment. A majority of the DEGs were most highly expressed at *t*_1_ or *t*_4_ (Table S6, Fig. S6B). Enrichment analysis revealed that many metabolic and cellular processes were overrepresented in the two fungi at *t*_1_. A small number of processes were enriched at the later time points, and the overrepresented processes exhibited time-dependent differences in the two fungi (Fig. S6C).

### Gene expression induced at the onset of SOM oxidation

**Fig. 3:**
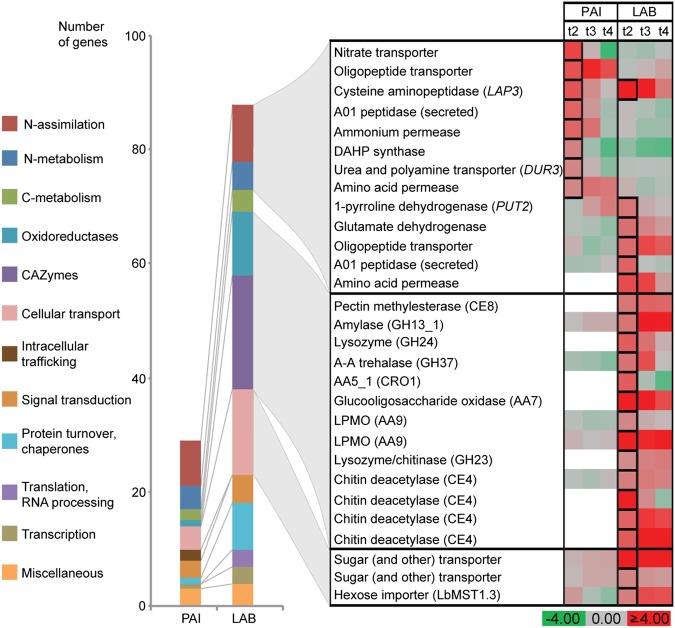
Expression of *P. involutus* (PAI) and *L. bicolor* (LAB) genes at the onset of SOM oxidation. The bar graph depicts the functional categories of annotated genes (29 in *P. involutus* and 88 in *L. bicolor*) that were among the top 20% most highly expressed genes at *t*_2_, and were upregulated more than twofold in pairwise *t*_2_ vs. *t*_1_ comparisons (*n* = 3, *p*_adj < _0.01, Wald test). The categories *CAZymes* and *Translation, RNA processing* were identified only in *L. bicolor*; *Intracellular trafficking* was only identified in *P. involutus*. Detailed information on the annotated genes is given in Tables S7 and S8. Fold-changes in expression for (*t*_2_–*t*_4_) vs. *t*_1_ comparisons, presented on a log_2_-scale, of selected genes from the *N metabolism*, *N assimilation*, *CAZymes*, *oxidoreductases*, and *cellular transport* categories are shown in the panel on the right. The *t*_2_ data points depicted in black squares indicate genes that were highly upregulated (≥twofold vs. *t*_1_) and expressed (top 20% at *t*_2_). Gene names given in parentheses in the *N metabolism* and *N assimilation* categories are homologs from *S. cerevisiae* that are significantly upregulated during ammonium limitation (Table S9). Procedures for identifying homologs of *P. involutus* and *L. bicolor* are described in Table S10; white color indicates that no homolog was identified

To identify the major transcriptional changes that occurred during the onset of SOM oxidation, the genes that were both highly upregulated (more than twofold) and highly expressed (among the 20% most highly expressed genes at *t*_2_) in pairwise comparisons of *t*_2_ and *t*_1_ samples were identified (Fig. 3). In both fungi, some of these genes were associated with N metabolism and the assimilation of organic N sources, and several shared sequence similarity with genes upregulated in *Saccharomyces cerevisiae* during ammonium limitation. However, the upregulated genes from the N metabolism and assimilation categories were different between *P. involutus* and *L. bicolor*. Upregulated genes in *P. involutus* but not in *L. bicolor* encoded nitrate, ammonium, and urea transporters. An upregulated amino acid permease-encoding gene of *L. bicolor* shared sequence similarity with several amino acid permeases of *S*. *cerevisiae*; close homologs of these *S. cerevisiae* genes were not found in *P. involutus* (Fig. S7).

The expression of genes encoding enzymes acting on carbohydrates (CAZymes) and auxiliary oxidoreductases (AAs) [52, 53] differed markedly between *P. involutus* and *L. bicolor* (Fig. 3). Genes encoding the following extracellular enzymes involved in the degradation of plant polysaccharides were highly expressed and upregulated in *L. bicolor*: pectin methylesterase (CE8), amylase (GH13_1), glucooligosaccharide oxidase (AA7), and two lytic polysaccharide monooxygenases (LPMOs, AA9). In addition, *L. bicolor* expressed several genes presumably involved in the degradation of microbe-derived material, including A–A trehalase (GH37), lysozymes (GH23 and GH24), and chitin deacetylases (CE4). Genes encoding these enzymes were either absent from the *P. involutus* genome or not upregulated at the onset of SOM oxidation. Further, the upregulated *L. bicolor* genes that encoded putative transporters were more numerous and diverse than those upregulated in *P. involutus*. The *L. bicolor* transporter genes included ones coding for the hexose import transporter LbMST1.3 [54] and several other transporters from the major facilitator superfamily (Fig. 3).

### Transcriptional responses to changing nutrient availability

**Fig. 4:**
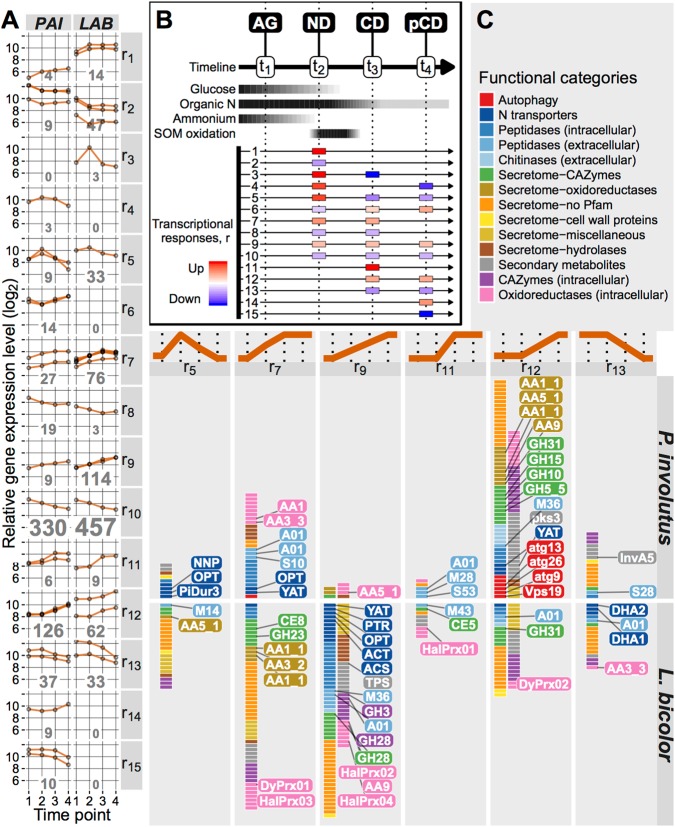
Transcriptional responses of ECM fungi to changing nutrient availability. **A** Main temporal gene expression profiles identified by clustering genes associated with metabolism and SOM-interaction, and differentially expressed in *P. involutus* (PAI, 1066 genes) and *L. bicolor* (LAB, 1120 genes) (Fig. S8). The main expression profiles were grouped into 15 distinct transcriptional response types (r_1_…r_15_) (panel **B**). The number of distinct genes within each response type category is indicated in each subpanel, with the font size proportional to the number of genes. **B** Identification of the transcriptional response types. Top, schematic representation of changes in glucose, organic N, ammonium-N, and SOM oxidation levels, determined by chemical analyses (cf. Figs. 1 and 2). AG active growth, ND ammonium depletion, CD glucose depletion, pCD prolonged glucose depletion. Bottom, observed patterns of qualitative changes in gene expression at the ND, CD, and pCD conditions relative to the preceding time point. These patterns were thus defining the 15 transcriptional response types. The response patterns are sorted according to the time point of expression change, prioritizing upregulation over downregulation, and positioning complementary pairs (up vs. down) next to each other. A missing box indicates a sustained expression level. **C** Genes clustered by virtue of their SOM-interaction annotation. Shown are the transcriptional response types that were specifically upregulated during ND (r_5_), upregulated during ND and CD (r_7_ and r_9_), sustained during ND and upregulated during CD and pCD (r_11_ and r_12_), and sustained during ND and downregulated during CD and pCD (r_13_). Functional gene categories are indicated by different colors and the boxes represent individual gene models. Genes discussed in the text are highlighted. See Dataset S1 for more detailed information

To characterize the major transcriptional changes that occurred over the time course of the entire experiment, genes that were coordinately expressed were identified (Fig. 4). A clustering procedure based on hierarchical functional annotations of genes was customized with the KEGG metabolism hierarchy and a novel hierarchy of genes associated with the decomposition of PCW derived polymers and assimilation of organic N (SOM-interaction genes), with a conservative statistical treatment to maximize the biological relevance (Figs. S8 and S9; Table S11). Transcriptional response type discovery was developed, and clusters of 15 types (r_1_… r_15_) were identified (Fig. 4A, B). Fourteen of these response types were found in *P. involutus* and 11 in *L. bicolor*. The largest number of genes followed the response type r_10_, i.e., highest expression during AG and a gradually decreasing expression over time. This expression pattern was observed for ca. 54% of the clustered genes in *P. involutus* (330 genes) and *L. bicolor* (457 genes), and was dominated by the metabolic pathway genes (Fig. S9A). Ten of the remaining response types represented genes that were upregulated during one or more time points associated with ND, CD, or pCD conditions (Fig. 4B bottom). These response types were dominated by SOM-interaction genes; functional annotations of the five most common types are shown in Fig. 4C (r_5_, r_7_, r_9_, r_11_, and r_12_). The functions and numbers of genes in these response types differed considerably in the two fungi: most genes in *L. bicolor* were upregulated in response to ND and CD/pCD; in contrast, most genes in *P. involutus* were only upregulated in response to CD or pCD. The temporal expression pattern associated with each response type was significant in these types as indicated by the enrichment of SOM-interaction genes for at least one of the species, or by the significantly limited metabolic genes with an expected amount of SOM-interaction genes (r_5_) (Fig. S9B).

In *P. involutus*, the number of SOM-interaction genes upregulated in response to ND was limited and included genes that encoded oxidoreductases: laccase-like multicopper oxidase (AA1), methanol oxidase (AA3_3), and copper radical oxidase (AA5_1 (CRO_2)). Some of the genes that encode extracellular peptidases (A01, S10) and N transporters (YAT and oligopeptide transporter (OPT) families) were similarly regulated (Fig. 4C, r_5_, r_7_, and r_9_). Temporary upregulation in response to ND (Fig. 4C, r_5_) characterized genes of three coordinated N transporters: OPT, and the previously identified urea (Dur3) and nitrate transporters (NNP) (cf. Fig. 3). The cohort of *P. involutus* genes that were upregulated in response to CD but not ND (Fig. 4C, r_12_) included several PCWDE genes, i.e., endoxylanase (GH10), glucoamylase (GH15), alpha glucosidase (GH31), endoglucanase (GH5_5), and LPMO (AA9); as well as two laccases (AA1_1) and a copper radical oxidase (AA5_1 (CRO3-5)). Other upregulated genes in this category encoded chitinases (GH18 and GH18–CBM5), intracellular and extracellular peptidases, and some N transporters. Notably, a cluster of six autophagy-related (atg) genes (atg9, atg13, atg15, atg17, atg26, and Vps19) was coregulated with the CD-responsive genes, suggesting the activation of a C starvation response [43]. The analysis also revealed temporal regulation of genes involved in the biosynthesis of the Fe^3+^-reducing metabolite involutin (InvA5 and InvD) [23, 55]. In terms of the response type, both genes showed sustained expression during AG and ND (Fig. 4C, r_13_).

In *L. bicolor*, genes upregulated in response to ND (Fig. 4C, r_5_, r_7_, and r_9_) included ones that encoded enzymes putatively involved in the oxidation of organic matter, including two laccases (AA1), aryl alcohol oxidase (AA3_2), and a copper radical oxidase (AA5_1 (CRO1)), which was also among the most highly expressed and upregulated genes at *t*_2_ (Fig. 3). The cluster analysis also identified several PCWDE genes upregulated by N, including the previously identified genes for pectin methylesterase (CE8) and amylase (GH13_1) (cf. Fig. 3), and, additionally, two pectinases (GH28), a beta-glucosidase (GH3), and an alpha-glucosidase (GH31). Several genes encoding intracellular peroxidases, including haloperoxidases of the heme-thiolate family (HalPrx01, HalPrx02, HalPrx03, and HalPrx04) and a dye-decolorizing peroxidase (DyPrx01) [13] were also up-regulated. Genes that were up-regulated or with unchanged elevated expression at ND included ones that encoded proteins involved in organic N assimilation: extracellular peptidases (A01, M14, M36, and M43) and a diverse set of N transporters (DHA1, DHA2, ACS, ACT, YAT, OPT, and PTR families) (Fig. 4C).

## Discussion

As demonstrated by recent studies, SOM extracted from the humic soil layer of a Norway spruce stand using hot water contains all major classes of biomolecules found in bulk SOM [13]. *P. involutus* and *L. bicolor*, when supplemented with glucose, have the capacity to decompose SOM by relying on oxidative mechanisms [13]. Here, using time-series spectroscopy and the same type of SOM extract, we show that SOM oxidation is linked to the liberation of organic N from SOM (Figs. 1 and 2). In both fungi, SOM oxidation was initiated when the readily available N source, i.e., ammonium, had been depleted. Following oxidation, organic N sources were utilized. In agreement with the findings of a previous study [21], the increased level of oxidation products declined when the fungi experienced C limitation. Therefore, despite their different evolutionary histories and foraging strategies, we observed functional convergence between the two species at the level of both SOM decomposition processes (SOM oxidation and liberation of organic N) and the regulation of SOM decomposition by similar nutritional signals, including limitation of inorganic N sources, presence of organic N source(s), and access to an energy source, e.g., glucose.

Sequential assimilation of N sources is common in fungi, with the ammonium utilized before other, less preferred, N sources [56, 57] and this was observed in our study as well. To examine whether *P. involutus* and *L. bicolor* sensed ammonium depletion, as has been observed in other fungi, we analyzed the regulation of genes homologs to ones that are upregulated during ammonium limitation in *S. cerevisiae*. Although most of those genes are present in the genomes of *P. involutus* and *L. bicolor*, and similar number of genes related to N-assimilation and N-metabolism were upregulated in both species, the types of upregulated genes differed markedly (Fig. 3). One gene that was upregulated in *L. bicolor* encoded an ammonium permease; this gene was located in a clade of fungal permeases, including *Gap1* (Fig. S7). In *S. cerevisiae*, *Gap1* is up-regulated during ammonium limitation and acts as a transceptor, i.e., both as a transporter and a receptor sensing the presence of amino acid substrates [58]. *P. involutus* and other related species from the Boletales examined here lack sequences in this clade. The genes upregulated during ammonium limitation in *P. involutus* included ones encoding inorganic N transporters and a *Dur3* homolog (*PiDur3*) [59], which encodes a plasma membrane transporter of urea and polyamines in *S. cerevisiae*.

The data presented in the current study indicated that SOM oxidation by *P. involutus* and *L. bicolor* proceeded in conjunction with hydrolysis. However, the components of the decomposition mechanisms and their regulation were distinct and different in the two fungi. The data suggested that SOM decomposition by *P. involutus* is a two-step mechanism of oxidation and hydrolysis, controlled by N limitation and C limitation, respectively. By contrast, SOM decomposition by *L. bicolor* is a one-step mechanism that involves a combined activity of oxidative and hydrolytic enzymes triggered by N limitation and sustained during C limitation.

Reduced iron (Fe^2+^) is required for the generation of hydroxyl radicals (∙OH) in the Fenton reaction [11]. Such iron was detected in the organic matter extract after incubation with *P. involutus* at the onset of SOM-oxidation (*t*_2_) using X-ray absorption spectroscopy. This coincided with the expression of genes encoding enzymes involved in the biosynthesis of the Fe^3+^-reducing metabolite involutin, InvA5 and InvD [55]. At this stage of SOM decomposition, only a small number of genes encoding extracellular enzymes were upregulated in *P. involutus*. However, at later time points and during CD, *P. involutus* expressed a larger number of extracellular hydrolytic enzymes, including proteases, chitinases, oxidases, and glycoside hydrolases (Fig. 4C). These genes were upregulated together with several genes associated with autophagocytosis, suggesting that *P. involutus* was undergoing C starvation response involving mycelial autolysis [43]. At the same time, the fungal biomass declined (Fig. 1A). Although some of the upregulated genes coded for extracellular enzymes that were probably involved in the degradation and assimilation of released cellular material, others (GH10, GH15, GH31, GH5_5, and LPMOs) most likely encoded enzymes involved in the decomposition of the PCW-derived material present in the SOM extract. The above observations suggest that ∙OH generation is to some extent temporally separated from the synthesis of extracellular (both oxidative and hydrolytic) enzymes. Evidence of such a temporal separation of the production of ∙OH and proteolytic enzymes was recently presented in a study examining the protein decomposition by *P. involutus* [60]. A temporally separated two-stage oxidation-hydrolytic mechanism was recently shown to be utilized during wood decay by the BR fungus *Postia placenta* [61].

In contrast with *P. involutus*, increased Fe^2+^ levels were not observed in the SOM extract of *L. bicolor* at any stage of the incubation. This suggested that the mechanism of SOM decomposition utilized by *L. bicolor* probably does not involve a nonenzymatic ∙OH oxidation. Furthermore, the *L. bicolor* biomass did not decrease and autophagy-related genes were not upregulated during C depletion, suggesting that the two fungal species respond differently to C starvation cues. Unlike in *P. involutus*, ND induced the expression of a number of *L. bicolor* genes associated with the decomposition of PCW derived polymers by saprotrophic fungi, including ones encoding LPMOs and CRO1, and genes associated with the decomposition of microbial products. Homologs of these genes were either lacking or not up-regulated in *P. involutus* (Fig. 3). Moreover, in *L. bicolor*, during C starvation, the expression of SOM decomposition genes upregulated during ND increased further, and a small set of additional genes, potentially also involved in SOM decomposition, was upregulated (Fig. 4C). The genes that responded to both ND and CD, or only to CD, included ones that are typically involved in the decomposition of PCW polymers by saprotrophic fungi, including pectin (GH28 and CE8), cutin (CE5), cellobiose (GH3), starch (GH13_1), small polysaccharides (AA7) [11], and cellulose (AA9), as well as the fungal cell wall component chitin (CE4). These genes also included ones coding for oxidative enzymes, such as DyP and HalPrx, which have been suggested to act on lignin-like compounds and participate in detoxification processes [62]. The observation that a gene of a hexose import transporter LbMST1.3 [54] was upregulated together with several PCWDE genes in *L. bicolor* (Fig. 3) suggests that this fungus indeed possesses some capacity to assimilate C released during SOM decomposition. Collectively, these data indicated that immediately after the onset of ND, a number of genes related to the enzymatic decomposition of PCW and microbial polymers were upregulated in *L. bicolor*, and continued to be upregulated during C depletion.

Studies have suggested that some of the remaining PCWDE genes seen in ECM fungi have been recruited for the modification of the PCW of the host during mycorrhizal formation [19, 20]. Here, we provide evidence that at least some PCWDE genes are upregulated in the absence of a host during N and/or glucose limitation as part of the SOM decomposition mechanisms of ectomycorrhizal fungi. This suggests that while during evolution of the ectomycorrhizal lineages the PCWD machinery got reduced [10], the remaining genes could have been either incorporated into the SOM decomposition mechanisms of ECM fungi or recruited as PCW modifying genes during mycorrhizal formation. Such a diversification of functions could happen even within a gene family. This hypothesis is supported by the fact that the endoglucanase GH5-5 gene from *L. bicolor* that was shown to participate in the remodeling of the PCW during mycorrhization [20] was expressed at low levels and not significantly regulated in our experiments.

The decomposition mechanisms of *P. involutus* and *L. bicolor* are distinctly different, in agreement with their diverse evolutionary origins, i.e., a BR wood decayer vs. litter-decomposing fungus [10]. In spite of these differences, the action of both mechanisms is controlled by N and C availability. This further suggests that the availability of photosynthetic products along with the type of available N in soil could act as a dual control over the decomposition activities of ECM fungi. That the host plant might actively control the decomposing activities of ECM fungi by controlling the amount of photosynthetic carbon provided to the fungus is suggested by soil microcosm experiments with *Pinus sylvestris* seedlings and ECM fungi including *P. involutus*. ^14^C pulse labeling of the seedlings showed that the amount of plant C allocated to the fungal mycelium was high at the early phase of colonization of litter patches, but the C flow dropped when these areas were fully colonized and the available nutrients were assimilated [63]. Alternatively, seasonality might control the decomposition activities of ECM fungi as suggested in field studies using enzyme assays [16, 64]. Further studies are needed to examine how the decomposition activities of ECM fungi are regulated when the fungi are growing in the field and in association with their plant host. Data generated from experiments in pure culture system as used in this study will provide the tools including the molecular biomarkers that could accurately predict the decomposition activities of ECM fungi in situ. Such markers must capture the action of both enzymatic and nonenzymatic (i.e., Fenton-based) reactions.

The impact of ECM fungi on soil carbon cycling remains controversial. Genomic comparisons have suggested that the decomposition potential of ECM fungi is much smaller than that of their saprotrophic ancestors, based on the gene losses for PCWDEs seen in ECM lineages [8, 10]. However, our results suggest that under nitrogen limitation ECM fungi oxidize SOM, while under nitrogen and/or carbon limitation some of the remaining PCWD related genes are upregulated. Whether these genes are used to only modify the SOM in order to further access entrapped nitrogen sources [65] or to release metabolic C is not clear, but the upregulated sugar transporter in *L. bicolor* suggests that some of this C can be assimilated by the mycelium. The impact of those PCWD related genes on C cycling processes might be considerable, particularly in deeper soil horizons where ECM species dominate [23]. Furthermore, ECM species might have an indirect impact on soil C cycling by affecting the availability of N. The upregulation of genes involved in the decomposition of proteins and other microbial-origin N compounds suggest that ECM fungi are able to access, decompose and assimilate organic N entrapped in SOM compounds. By doing so, ECM fungi may induce or potentiate N limitation of free-living, saprotrophic microbial decomposers, which may impede the soil C cycling and increase soil C storage [66, 67].

## Supplementary information

Supplementary Information

Dataset S1

## Data Availability

The chemical and spectral datasets (Py-GC/MS, XANES, FTIR) generated and analyzed during the current study are available from the corresponding author on reasonable request. The RNA-Seq datasets generated and analyzed during the current study are available in the Gene Expression Omnibus (GEO) repository, https://www.ncbi.nlm.nih.gov/geo/query/acc.cgi?acc=GSE110485.
