# Supplementary material for: The soil organic matter decomposition mechanisms in ectomycorrhizal fungi are tuned for liberating soil organic nitrogen
Source: ISME J. 2018 Dec 11;13(4):977–88. doi: 10.1038/s41396-018-0331-6 (PMC6461840; doi:10.1038/s41396-018-0331-6)
Supplement: Supplementary file 1 — Supplementary Information [file 41396_2018_331_MOESM1_ESM.docx]

**Supplementary Information**

**Article title:** The soil organic matter decomposition mechanisms in ectomycorrhizal fungi are tuned for liberating soil organic nitrogen

**Authors:** César Nicolás, Tomas Martin-Bertelsen, Dimitrios Floudas, Johan Bentzer, Mark Smits, Tomas Johansson, Carl Troein, Per Persson and Anders Tunlid

**Materials and methods**

**Culture Conditions.** Cultures of *P. involutus* (Batsch Fr., ATCC 200175) and *L. bicolor* (Maire P.D. Orton) were grown (18 ^o^C, in the dark) on a layer of glass beads in Petri dishes containing a liquid minimum Melin-Norkrans (MMN) medium for 9 and 18 d, respectively (1). The MMN medium consists of 2.5 g l^–1^ glucose, 500 mg l^–1^ KH_2_PO_4_, 200 mg l^–1^ NH_4_Cl, 150 mg l^–1^ MgSO_4_·7H_2_O, 25 mg l^–1^ NaCl, 50 mg l^–1^ CaCl_2_, 12 mg l^–1^ FeCl_3_·6H_2_O and 1 mg l^–1^ thiamine-HCl; pH 4.0. After 9 days of incubation (18 °C in the dark), the medium was replaced with MMN medium without N to induce an N-deprived mycelium (2). After 24 h, the mycelium was washed in sterile water, and the SOM extract (10 ml) was added.

SOM was extracted from the upper 10-cm soil layer in a 61-y-old Norway spruce stand growing in a N-poor site in central Sweden (soil pH, 5.0) using hot water (3). Particles were removed by filtration (0.2 µm), and low molecular weight metabolites were partly removed by ultrafiltration (cut-off, 1 kDa). The concentration of the SOM extract was adjusted, and the extract was supplemented with glucose to a final concentration similar to that in the MMN medium (1).

**Chemical Analysis.** Total organic carbon concentrations were measured using a TOC Analyzer (Shimadzu, Kyoto, Japan), and total nitrogen (TN) using the same apparatus with a TNM-1 detector. Glucose concentration was measured using the glucose (GO) assay kit (Sigma-Aldrich, Seelze, Germany). Nitrate and ammonium concentrations were determined using flow injection analysis (FIAstar 5000 Analyzer, FOSS, Denmark).

Pyrolysis gas chromatography-mass spectrometry (Py-GC/MS) was conducted on freeze-dried organic matter extracts. Samples were analyzed using a TurboMass/ Autosystem XL with Frontier Lab double Shot pyrolyser (Perkin-Elmer, Waltham, MA, USA). Pyrolysis data were acquired and processed with QCalibur 1.4 SR1 software (Thermo Finnigan, San Jose, CA, USA), and peaks were identified by comparison with published and stored data (NIST library). The ratio of 4-acetylguaiacol to *trans*-propenylguaiacol(Ox/C3-G) was used as a marker of the degree of side-chain degradation of lignin (4).

The Fe K-edge XANES spectra were collected at beamline I811, MaxLab, Lund, Sweden. The beam photon flux on the sample was 10^11^-10^12^ photons per second and the beam size on the sample was 0.5 x 0.5 mm^2^. The beam was monochromatized with a double-crystal Si(111) monochromator, detuned to 60% of the maximum intensity for rejection of higher order harmonic radiation. A reference Fe metal foil was used as an internal energy calibration of the monochromator for all spectra; the first inflection-point of the main edge of the metal foil was set at 7 111.08 eV. The spectra were recorded in continuous scan mode from 6 900 to 7 150 eV and between 2 and 10 scans were averaged per sample. The X-ray absorption spectra were processed and analyzed using the software SIXPack (5). The calibrated spectra were pre-edge baseline corrected and normalized at the post-edge to 1. In order to obtain the redox state of Fe, the pre-edge peak was examined as described by Wilke et al. (6). The centroid position of this peak is indicative of the Fe redox state. A spline baseline was adjusted manually and the pre-edges were deconvoluted into Gaussian components using the software Fityk (version 0.9.8) (7). The integrated area and the centroid (if fitted with more than one function) were calculated and one-way ANOVA was used for the statistical analysis of the centroids to establish differences between treatments.

The N K-edge XANES spectra (390-430 eV) were collected at the beamline 11ID-1 (SGM), Canadian Light Source, Saskatoon, Canada. In order to measure the sample, the organic matter extract was poured on indium foil and dried at 30 °C. The indium foil was then fixed to sample the holder using double-sided carbon tape. To avoid sample damage, every scan was recorded in a fresh, unexposed spot. Ammonium sulphate was used for energy calibration, and normalization was accomplished by measuring the beamline flux (I_0_) with an in-line gold mesh. The main peaks of the XANES spectra were assigned by comparison with spectra of model compounds found in the literature (8). The N K-edge peaks corresponded to 1s →π* transitions were assigned to the following compounds: (a) non-peptide C=N as in aliphatic imines and/or in aromatic pyridines, pyrazines, pyrimidines (399.2 eV); (b) nitriles and/or aromatic systems such as purine, pyrazole and/or imidazole (400.2 eV); (c) amide/amine N (401.5 eV) and (d) N-heterocyclic aromatics such as pyrrole (403 eV). The strong peak at 406 eV was assigned to σ* transitions in inorganic N of ammonium and other N moieties. The calibrated N K-edge spectra were pre-edge baseline corrected and normalized at the post-edge to 1. Then, the spectra were fitted using Gaussian and error functions with fixed energy centers and full width at half maximum using the software Fityk (version 0.9.8) (7). The relative abundances of the π* transitions were finally determined with respect to the sum of all π* transitions and were used as indicators of the content of major N types in the samples. In order to semi-quantitatively compare the spectra, an ordination method (principal component analysis) based on the relative abundance of π* transitions (a, b, c and d) was done to find differences in the changes of organic N speciation between fungi.

Multivariate curve resolution-alternating least squares (MCR-ALS) analysis (9,10) was used to decompose the infrared spectral intensities into two matrices: C that contains the concentration profiles of the chemical species and S^T^ that contains the pure spectra; plus a residual matrix. The MCR-ALS required firstly the determination of the number of components and an initial estimate of either concentration (C) or spectra (S^t^). Since MCR-ALS is an iterative approach, a factorization of the data matrices using singular value decomposition was used to estimate the initial number of components and an evolving factor analysis to find suitable initial estimates. By the alternating least square algorithm, the concentration c and the spectra were then estimated until convergence is achieved by minimizing the error criteria of the sum of the squared residuals. The possible solutions were limited by constraining to non-negativity and unimodality in the concentration profiles and normalization in the spectra of matrix S^t^. In case of *L. bicolor*, the unimodality was removed.

**Transcriptome Analysis.** FastQC (ver. 0.11.2) (11) was used for quality control of raw RNA-Seq reads (single-end), and ribosomal RNA-like sequences were removed using riboPicker (standalone ver. 0.4.3, SILVA database ver. 119) (12). Filtered gene models and repeat masked genome assembly (scaffold) sequences were retrieved from the Joint Genome Institute (JGI) MycoCosm database (13) (*P. involutus* ver. 1.0, *L. bicolor* ver. 2.0). RNA-Seq reads were aligned against the transcriptome using TopHat2 (ver. 2.0.13) (14) with Bowtie2 (ver. 2.2.3.0) (15) and the following parameters: --transcriptome-only -x 1 -I 5000 --library-type 'fr-firststrand'. SAMtools (ver. 0.1.19) (16) was used to sort and convert the BAM files to SAM files. Transcript abundances were measured as read counts in CDS using HTSeq (ver. 0.6.1) (17) with the following parameters: -s 'reverse' -t 'CDS' -i 'gene_id' -m 'intersection-nonempty'. Genes with mean count below 11 across all samples were removed. Read count normalization factors were calculated for each fungus using EDASeq (ver. 2.8.0) with 50 strata and full quantile normalization to correct for transcript length and G/C-content within each sample (18), and combined with sample size factors to adjust for different sequencing depths using DESeq2 (ver. 1.14.1) (19). Principal component analysis (PCA) was performed on normalized and regularized log-transformed (DESeq2) RNAseq data blinded to the experimental design for quality assurance.

**Differential Expression Analysis.** To identify genes with significant expression level changes over the whole time course, a likelihood-ratio test was used to compare a constant generalized linear model (GLM) to a full GLM with coefficients for all time points. Genes were identified as differentially expressed over the whole time course (“time-DEGs”) if the full GLM gave a better fit, at false discovery rate (FDR) of 1%. A minimum expression level threshold was automatically applied (DESeq2).

For each time-DEG, robust average log_2_ expressions for all four time points were extracted from the fitted GLM. Genes were grouped by the time point of max expression (Table S6). These groups were analyzed with GOseq (ver. 1.26.0) for enrichment of KOG and KEGG categories using all gene models encoded in the genome as background while adjusting for the length bias which may affect RNA-Seq data (20). Differentially expressed genes (DEGs) from pairwise comparisons of t_2_, t_3_ and t_4_ against t_1_ were identified using the Wald-test in DESeq2. *“*Highly expressed and upregulated genes” for each pairwise comparison was defined as those of the top 20% most highly expressed genes and that were differentially expressed (FDR = 1%) and at least two-fold upregulated. *P*-values were adjusted for multiple testing by calculating the false discovery rate (21). R version 3.3.2 was used throughout all bioinformatics analyses (22).

**Co-expression analysis.** To identify the main temporal gene expression patterns, clusters of tightly co-expressed genes with a shared biological annotation were identified using the FunPat tool (23). FunPat first finds clusters within each annotation category (gene set) to obtain gene set patterns. These are clustered across annotation categories into main patterns. Each gene expression profile is thus first projected to a gene set pattern profile which in turn is projected to a final representative main temporal expression pattern, which was used as the basis for grouping of genes into qualitative response types. FunPat leaves some genes unclustered (Singletons); these were excluded from the clustering results and not used for the response type classification (described below).

FunPat exploits annotation categories organized into a hierarchy according to specificity to prioritize identification of co-expressed genes associated with the most specific function. We built a customized annotation hierarchy by combining distinct annotation sources and manually curated categories for a targeted co-expression analysis: The annotation hierarchy of KEGG metabolism pathway genes and a novel annotation hierarchy of SOM interaction genes (Fig. S8A).

By focusing on highly correlated clusters of genes within each annotation category, we expect to extract the most robust and functionally relevant expression patterns while disregarding genes not clustered by FunPat (Singletons) as these are not highly correlated to any other functionally related gene. To reduce spurious clusters and increase biological relevance, the clustering procedure was based only on the annotated time-DEGs with strongest signal and constrained within the selection of a priori known biological function annotation categories. The robust average log_2_ gene expressions from the GLM were used as input together with reconstruction error variances according to formulas in Love et al. (19).

Default FunPat parameter settings were used with some exceptions:

- *alphacorr*: The statistical assessment of a pattern profile against a constant expression pattern was set to be controlled at a FDR of 10%, as the default (1%) was deemed too stringent by not clustering most genes.
- *p.sel*: The Ranking Score used within FunPat for the order (low-to-high) of genes initializing the iterative fitting algorithm was determined not by *p*-values (default) but by absolute fold changes vs. AG, using the reciprocal of the highest fold change value.
- *seed* and *candidate genes* were not defined from *p*-values alone, as detailed below.

”Seed” genes have a leading role in the FunPat clustering algorithm and were here defined as those time-DEGs with more than two-fold expression level change up/down at any time vs. AG (t_1_). A less differentially expressed gene (candidate) may still contain relevant biological signal and is redeemed by the FunPat clustering and gene selection procedure if its expression profile is highly similar to a seed gene with the same annotation. Since the bulk of annotated genes decrease in fold changes for increasing *p*-values, the biologically relevant signal seems to disappear above some *p-*value threshold (Fig. S8B, insert). The threshold was thus set where the bulk of genes had less than two-fold expression change. Accordingly, for both species, only genes from the top 1 000 most significant time-DEGs were used as candidate genes for the FunPat procedure (Fig. S8B).

The identified main temporal expression profiles were grouped into qualitative response types based on first and second order differences defined at each time point:

$$\frac{\Delta x}{\Delta t}\left( t_{i} \right)=x_{i}-x_{i-1}\text{; 0 for }i\in\left\{ 1,n+1 \right\}$$

$$\frac{\Delta^{2}x}{\Delta t^{2}}\left( t_{i} \right)=\frac{\Delta x}{\Delta t}\left( t_{i+1} \right)-\frac{\Delta x}{\Delta t}\left( t_{i} \right)$$

The $n=4$ time point index values $t_{i}=i=1,\ldots,n$ were used instead of the actual elapsed number of days; *x* denotes a gene expression profile projected to a main pattern on log_2_ scale.

To qualitatively describe dynamical patterns, three possible states were defined for each of these first and second order differences: < 0, 0, and > 0. Together with the time point of max expression, these qualitative states encoded a temporal response pattern. Since an exact 0 is rarely obtained, tiny differences were disregarded as biologically uninteresting and approximated by zero, such that expression profiles that were visually similar on a relative scale were assigned the same response type. The time point of max expression was defined by the max of the cumulative sum of the modified first order differences (the earliest occurring if non-unique).

A tiny relative change, $\epsilon$, was defined in terms of the constant rate of expression change required throughout the entire time course to span the whole range of *x*:

$$\epsilon=\frac{{\max_{i}x}_{i}-\min_{i}x_{i}}{t_{4}-t_{1}}s,$$

where $s=0.9$ is a scaling parameter. The value of $\epsilon$ was capped at $\epsilon_{\text{max}}=0.45$. When $\left| \frac{\Delta x}{\Delta t} \right|<\epsilon$, the first order difference was set to zero, and then the second order difference was computed. Similarly, a tiny second order difference was approximated by zero, while ensuring consistency by keeping a non-zero value if the modified $\frac{\Delta x}{\Delta t}$ changed sign. The parameters were tuned from the criterion that the most frequent main expression patterns (i.e. a constant gradual decrease) should be assigned to an identical response type encoding this qualitative pattern for both fungi (r_10_).

To enable detection of slowly but consistently drifting expression levels that would otherwise be considered as tiny changes at each time point, drift-intervals were identified by searching for consecutive time points of consistent increase or decrease with a cumulative change larger than $1.5\epsilon_{\text{max}}$. The first order differences at all the time points within the drift-interval were replaced with their mean to induce zero-valued second order differences while noting the steady non-zero change.

Statistical significance of the identified response patterns within each main annotation category was assessed by a permutation test on the time points along the lines of Ernst et al. (24) (see below). The algorithm devised for response type classification and the permutation test were implemented in R (22). Scripts are available upon request.

**Identifying significant response types.** Neither the differential expression analysis nor the FunPat clustering procedure makes use of the temporal ordering of the data points; the time points are considered as independent conditions in those parts of the transcriptome analysis. Response type classification is an important additional analysis step because it directly exploits the temporal ordering of the samples. It is therefore vital to make statistically valid claims of whether the observed response types are significant temporal expression profiles not due to random chance. Following Ernst et al. (24), our null hypothesis is that the data is memoryless and observed response pattern profiles are a result of random fluctuation in the gene expression values.

Permutations of the four time points were used to simulate the null distribution. For each of the 4! = 24 permutations, the main pattern expression values in the FunPat output were permuted accordingly and the response type for each clustered gene was identified. Note that other response types may be identified from the permuted data than those presented in Fig. 4 in the main manuscript.

Since each gene is assigned to exactly one response type (a few exceptions exist which we ignore in the analysis) we follow Ernst et al. (24) and assume that, under the null hypothesis, the number of genes in each response type is distributed as a binomial random variable. The expected number of genes varies between the response types. To calculate a *p-*value of enrichment (or depletion) for genes in each response type, we focused on genes clustered by FunPat through annotation categories within the two main annotation categories: metabolism or SOM interaction (cf. Fig. S8A).

Since FunPat clusters have a minimal size of 3 genes (default sizecl parameter), this is the minimal number of genes observable in a response type. When a given response type is not detected in a specific permutation, we cannot know whether 0, 1, or 2 genes should have been assigned to the response type according to the null. A censored version of the log-likelihood function was therefore implemented based on <https://stats.stackexchange.com/questions/19804/simple-case-of-mnar-missing-data#19842> and optimized to find maximum likelihood estimates (MLE) of the probability parameter in the binomial distribution for each response-type/main-annotation-category combination. In contrast to Ernst et al., our setup does therefore not allow us to compute the MLE from the mean over number of genes across permutations divided by a fixed gene total. Instead, we optimized (numerically) the censored log-likelihood expression directly.

P-values of enrichment and depletion for genes were computed in both species for all 15 response types identified in the original (unpermuted) data. To correct for multiple testing of these 60 hypotheses, the error rate of any false rejection (family-wise error rate) was controlled at 5% using Holm’s sequentially rejective Bonferroni test (25).

**Annotations.** KOG annotations and protein sequences were retrieved from JGI MycoCosm database (13). Gene models encoding peptidases (searching against peptidase full-length sequences in MEROPS ver. 10) (26), enzymes active on carbohydrates (CAZymes) including auxiliary redox activities (AAs), peroxidases, tyrosinases and multidomain natural product-biosynthesis enzymes were annotated as previously described (27). Nitrogen (N) transporters were identified by using annotation from the genome of *L. bicolor* (28). Genes encoding enzymes in metabolic pathways were identified using the BlastKOALA tool (29) removing nonsense pathways. Secondary metabolite gene clusters were predicted using the antiSMASH tool (ver. 3.0.4) (30) (Table S1 and S2). Prediction of secreted proteins was made from a custom pipeline of well-known prediction tools (31-40) (Fig. S1).

To identify genes in *P. involutus* and *L. bicolor* potentially encoding proteins that are homologs to those being upregulated in *Saccharomyces cerevisiae* during ammonium limitation, we collected a list of 41 proteins in *S. cerevisiae* that are significantly upregulated during N-starvation conditions (41, 42). The list was used as a query to identify putative homologs in the genomes of *P. involutus* and *L. bicolor* (Joint Genome Institute, genome.jgi.doe.gov) using BLASTP search, cut-off < 4.00E-20). In *P. involutus*, we identified 39 putative homologs of which 12 were significantly upregulated whereas 38 were identified and 8 upregulated in *L. bicolor* (Table S9).

One-to-one orthologous proteins were predicted using reciprocal best alignment heuristic as implemented in Proteinortho (ver. 5) using the conserved synteny option (43).

**Phylogenetic Analysis.** A preliminary dataset of all gene catalog proteins carrying the PF00324 domain was assembled from eight Agaricomycotina genomes (MycoCosm) (13). A set of characterized sequences from *S. cerevisiae* and *Hebeloma cylindrosporum* (28) the Yeast Amino Acid Transporter (YAT) family was also included in the dataset. A preliminary alignment was constructed using MAFFT (44). The alignment was examined, and poorly predicted or annotated proteins were removed from the dataset (Hydpi-2 91335, 28952, 184859, 169849; Agabi-varbisH97-2 176452, 184341; Amamu-1 535075, 185765; Gymlu-1 76583; Plicr-1 26265, and Paxin-1 60036). From that alignment, proteins belonging in the YAT clade were detected and a new, smaller dataset was generated. A new alignment of the YAT dataset using the strategy FFT-NS-i was generated and after examination of the alignment, when possible, some gene catalog models were replaced by better quality ones (see Fig. S6). The final alignment was examined and poorly aligned regions were removed using Jalview (45). A phylogenetic analysis was performed using RAxML (46) at Cipres (47) under the model PROTCATWAG, with 200 bootstrap runs and the sequence HNM1-YGL077C as outgroup.

**Supplementary figures, tables and dataset**

**Figure S1.** Identification of secreted proteins.

**Figure S2.** Changes in total organic C content and pH of the soil organic matter extract.

**Figure S3.** Chemical modifications of the soil organic matter extract during decomposition as revealed by FTIR spectroscopy.

**Figure S4.** X-ray absorption spectroscopy at the Fe K-edge of the soil organic matter extract.

**Figure S5**. X-ray absorption spectroscopy at the N K-edge of the soil organic matter extract.

**Figure S6.** Global transcriptional changes in *P. involutus* and *L. bicolor* during the decomposition of the soil organic matter extract.

**Figure S7.** RAxML phylogenetic analysis of the YAT family (amino acid permeases).

**Figure S8**. Annotation scheme and selection of genes used in the FunPat co-expression analysis.

**Figure S9.** Temporal co-expression profiles and response type significance.

**Table S1.** Identification of secondary metabolite biosynthesis gene clusters in the *P. involutus* genome using antiSMASH.

**Table S2.** Identification of secondary metabolite biosynthesis gene clusters in the *L. bicolor* genome using antiSMASH.

**Table S3.** Vibrational frequencies from FTIR spectra and assignments for the soil organic matter extract.

**Table S4.** Organic N composition of the soil organic matter extract incubated with *P. involutus* and *L. bicolor.*

**Table S5.** Statistics of RNA sequencing (RNA-Seq).

**Table S6.** Differentially expressed transcripts in *P. involutus* and *L. bicolor* during growth on the soil organic matter extract.

**Table S7.** Functional features of the most highly up-regulated and expressed genes at the onset of SOM oxidation in *P. involutus*.

**Table S8.** Functional features of the most highly up-regulated and expressed genes at the onset of SOM oxidation in *L. bicolor*.

**Table S9.** Genes upregulated during ammonium limitation in *Saccharomyces cerevisiae*.

**Table S10.** Identification of homologs displayed in Fig. 3.

**Table S11.** Response types significance testing.

**Dataset S1.** Annotation of genes in FunPat cluster from response types shown in Fig. 4C (separate Excelfile).

**
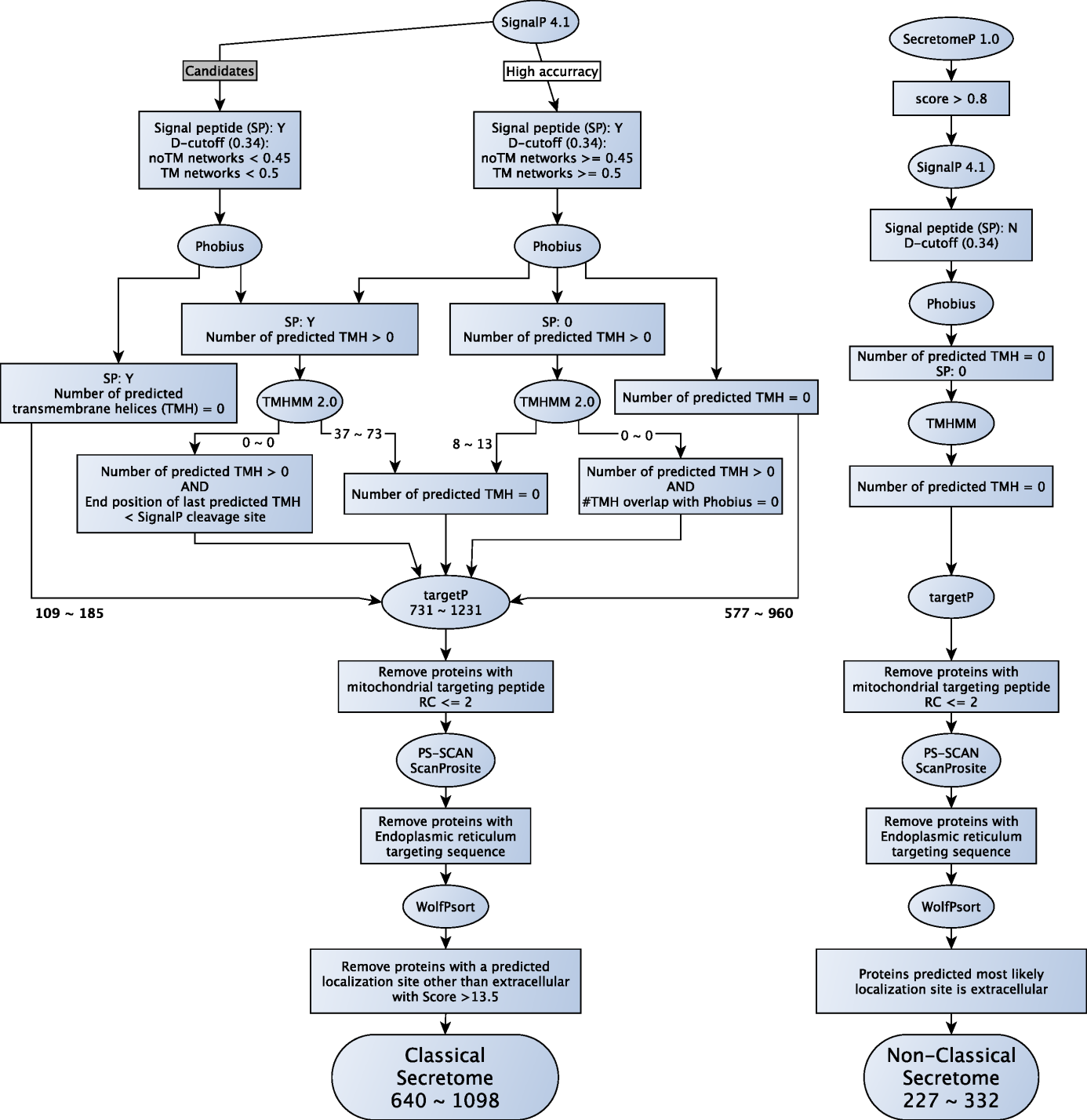
Figure S1.** Identification of secreted proteins. Classically and non-classically secreted proteins are predicted by two pipelines combining published prediction tools. Ellipses signify a tool; rectangles contain the filtering criteria on the output from the tool pointing into the rectangle. Numbers along arrows indicate the subtotals of predicted secreted proteins from the gene models of each species (*P. involutus*~*L. bicolor*); final number of genes in the predicted potential secretome are at the bottom of the scheme. TMH: Trans-Membrane Helix.

**Figure S2.** Changes in total organic C content (A) and pH (B) of the soil organic matter extract incubated with *P. involutus* (left plot) and *L. bicolor* (right plot). Bars indicate ±SE (*n*=3).


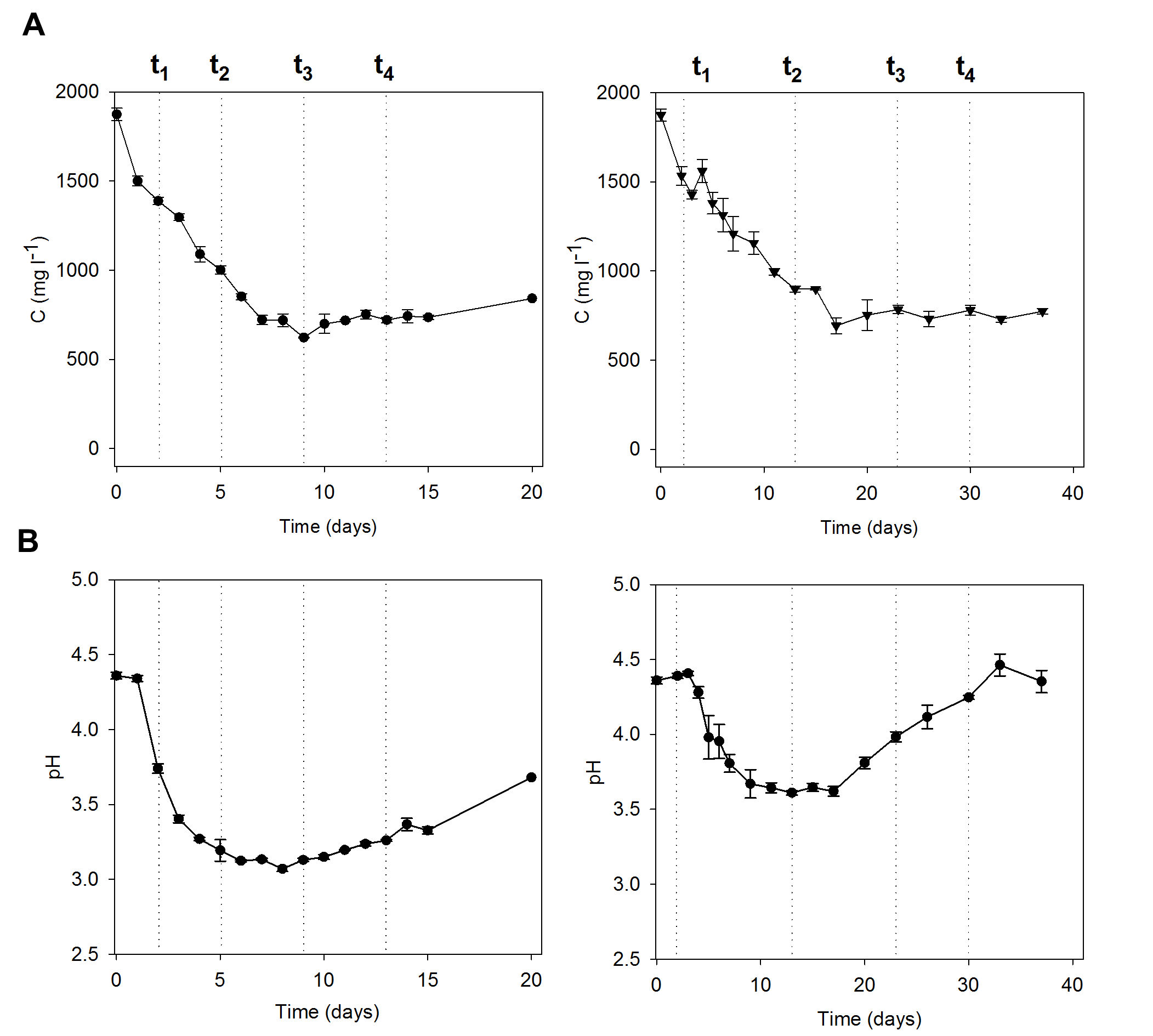


**Figure S3.** Chemical modifications of the soil organic matter extract (SOM) during decomposition by *P. involutus* (left plots) and *L. bicolor* (right plots) as revealed by FTIR spectroscopy*.* (A) Changes in the FTIR spectra of the SOM extract. All spectra have been normalized to the same total area over the wavenumber region displayed (*n* = 3).“t_0_” indicates the spectrum of the SOM extract before incubation with the fungus. The time point t_1,_ t_2,_ t_3_ and t_4_ are defined in Fig. 1 (main text). (B) Concentration profiles of four spectral components identified by Multivariate Curve Resolution analysis (MCR-ALS). (C) Resolved FTIR spectra of the components obtained from MCR-ALS analysis.


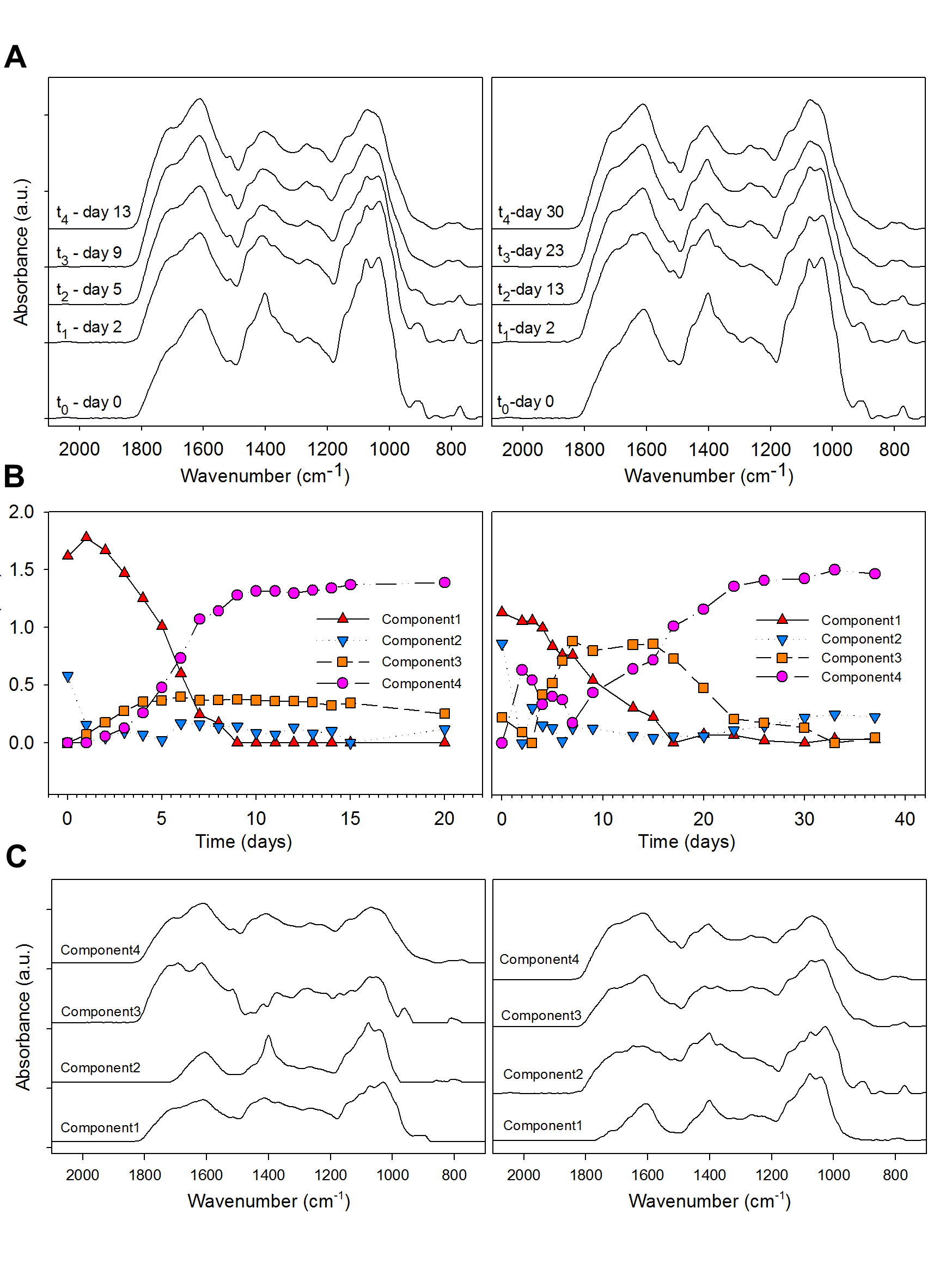


**Figure S4.** X-ray absorption spectroscopy at the Fe K-edge of the soil organic matter (SOM) extract. Shown are representative spectra obtained at the beginning of the experiment (SOMt_0_) and incubated with *P. involutus* during 5 days (PAIt_2_). On the left, the normalized absorption spectra, with the inset indicating the pre-edge region used to estimate the iron oxidation state. On the right, the selected normalized pre-edge spectra for SOMt_0_ (top) and PAIt_2_ (bottom) are shown. Vertical dashed lines indicate iron oxidation states as indicated by Wilke et al. (6), and the vertical solid line indicates the position of the centroid of the sample.


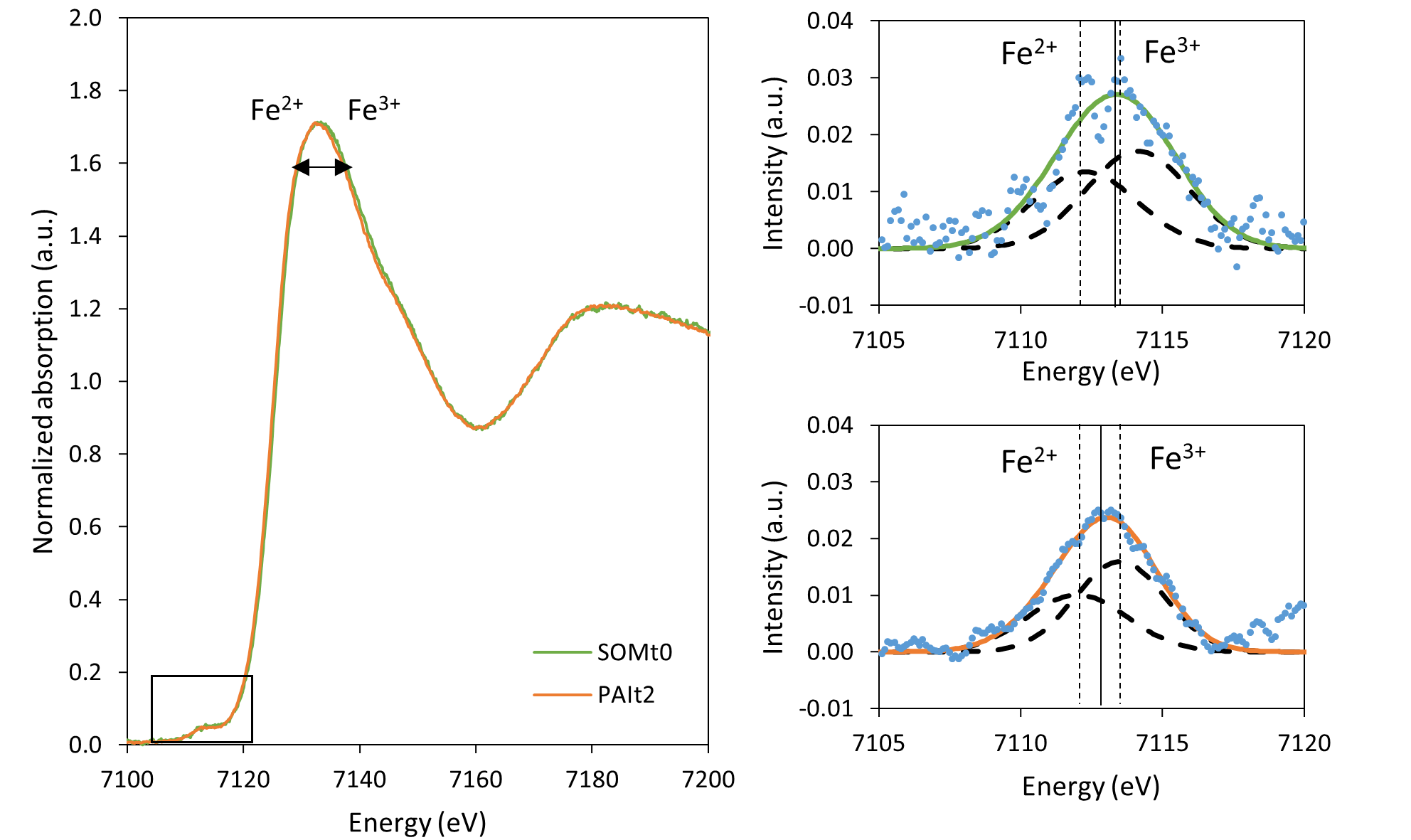


**Figure S5.** X-ray absorption spectroscopy at the N K-edge of the soil organic matter (SOM) extract. (A) X-ray absorption spectra at the N K-edge of the organic matter extracts decomposed by *P. involutus* (left plot) and *L. bicolor* (right plot) along the incubation. Vertical lines indicate types of N: non-peptide C=N as in aliphatic imines and/or in aromatic pyridines, pyrazines, pyrimidines (399.2 eV); (b) nitriles and/or aromatic systems such as purine, pyrazole and/or imidazole (400.2 eV); (c) amide/amine N (401.5 eV) and (d) N-heterocyclic aromatics such as pyrrole (403 eV). (B) Representative deconvolution of N K-edge spectra of the organic matter extract. Deconvoluted peaks correspond to the heterocyclic-N (399.2 eV), nitriles and aromatic-N (400.2 eV), amidic-N (401.5 eV), pyrrolic-N (403 eV), σ resonances (405.4, 406.6 and 412.5 eV), and the error function step (403.7 eV).


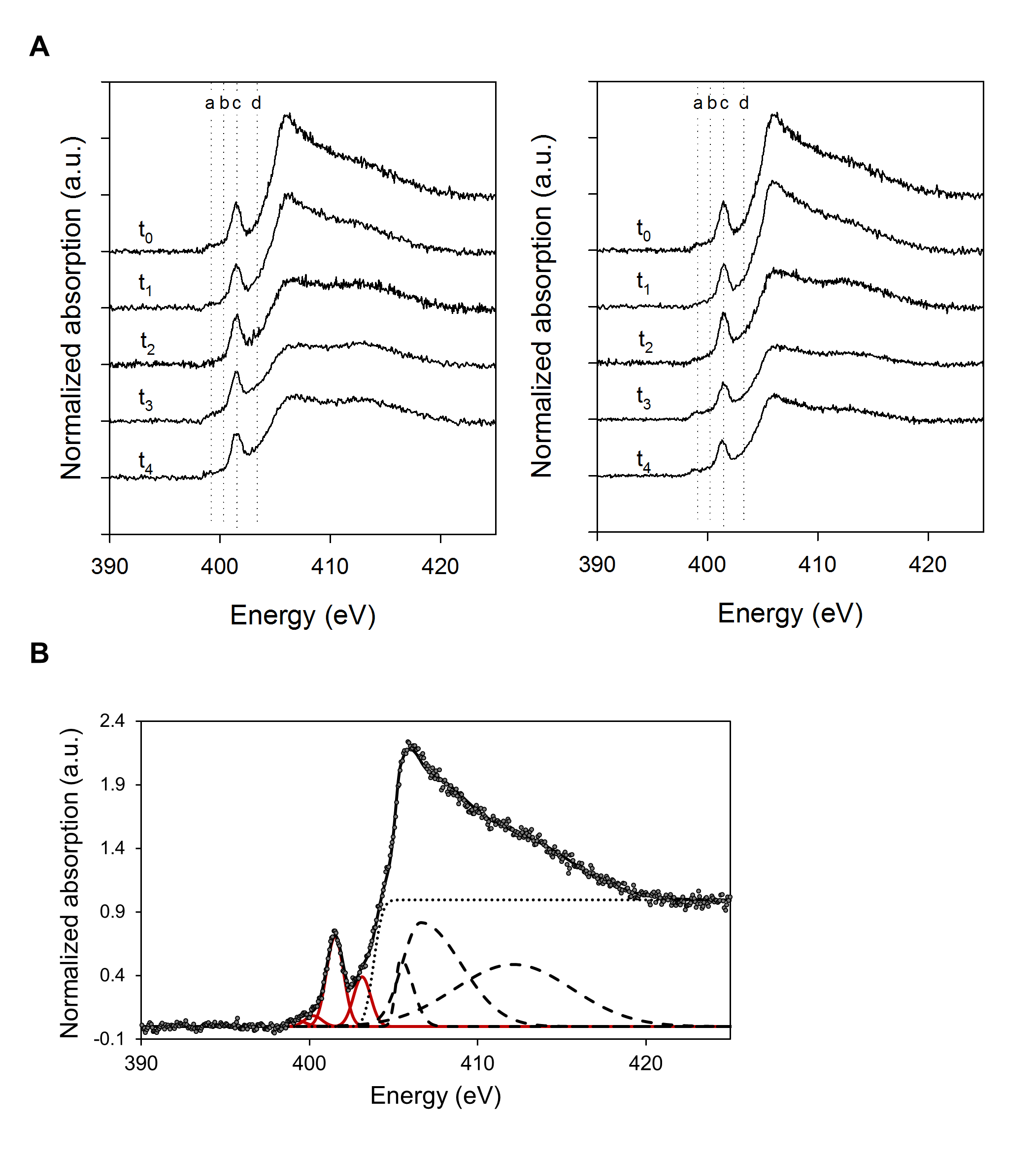


**Figure S6.** Global transcriptional changes in *P. involutus* (PAI) and *L. bicolor* (LAB) during the decomposition of the soil organic matter extract. (A) Principal Component analysis (PCA) performed on the expression levels of 12103 transcripts in PAI and 14472 in LAB. Each point in the PCA represents a replicate (*n*=3)*.* (B) Number of proteins in the KOG category “Metabolism” encoded by the most highly and differentially expressed genes at t_1,_ t_2,_ t_3_ and t_4_. (C) KOG enrichment analysis of the genes that were most highly expressed at t_1_, t_2,_ t_3_ and t_4,_ respectively. Shown are the KOG categories that are significantly over-represented (*p*_adj_< 0.01) for at least one of the time points. The scale bar shows the adjusted *p*-values of enrichment for all *p*_adj_ ≤ 0.20.

**
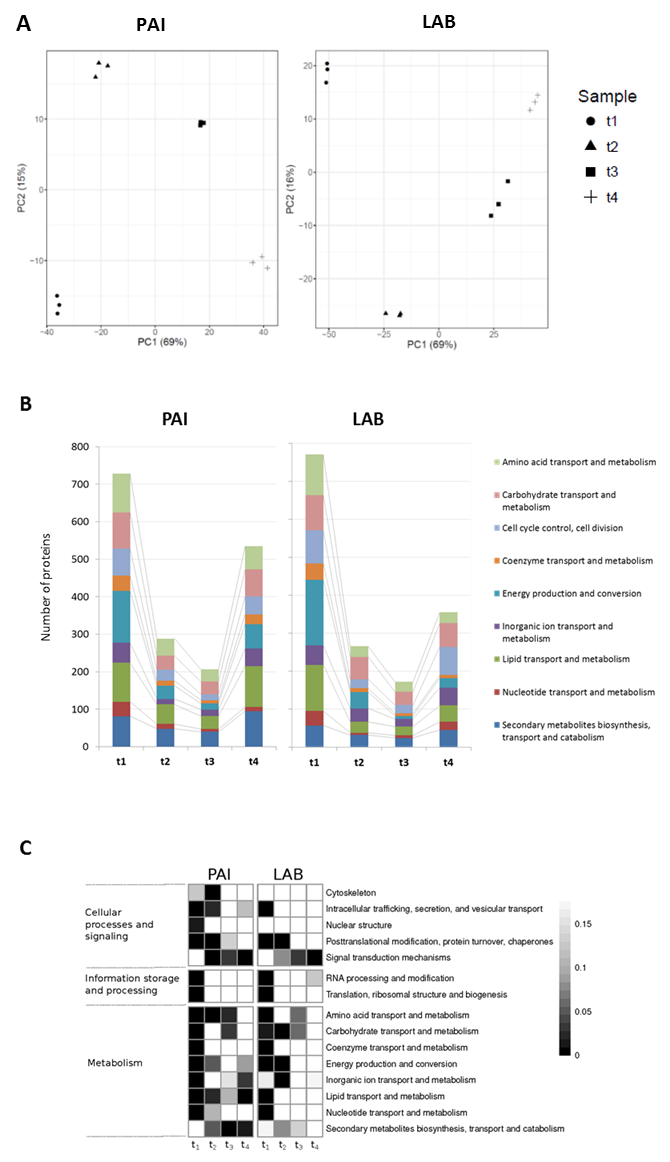
**

**Figure S7.** RAxML phylogenetic analysis of the Yeast Amino Acid Transporter (YAT) family based on predicted proteins from genomic data across 8 Agaricomycotina genomes. Characterized YAT proteins of *Saccharomyces cerevisiae* and *Hebeloma cylindrosporum* are highlighted with a black circle. The ACT protein from *S. cerevisiae* was used as outgroup. *Paxillus involutus* and *Laccaria bicolor* sequences are highlighted in red and blue, respectively. Numbers in parenthesis next to a protein ID indicate the ID of the protein model found on the gene catalog (GC) of the corresponding genome at the JGI database. The assignment of clades to groups I, II, and III was based on Lucic et al. (28). Arrows indicate the up-regulated permease genes shown in Fig. 3. Bootstrap values equal to or above 70 are shown. Acronyms: Amamu: *Amanita muscaria*, Agabi_varbis: *Agaricus bisporus var. bisporus*, Conpu: *Coniophora puteana*, Gymlu: *Gymnopus luxurians*, Hydpi: *Hydnomerulius pinastri*, Plicr: *Plicaturopsis crispa*

**
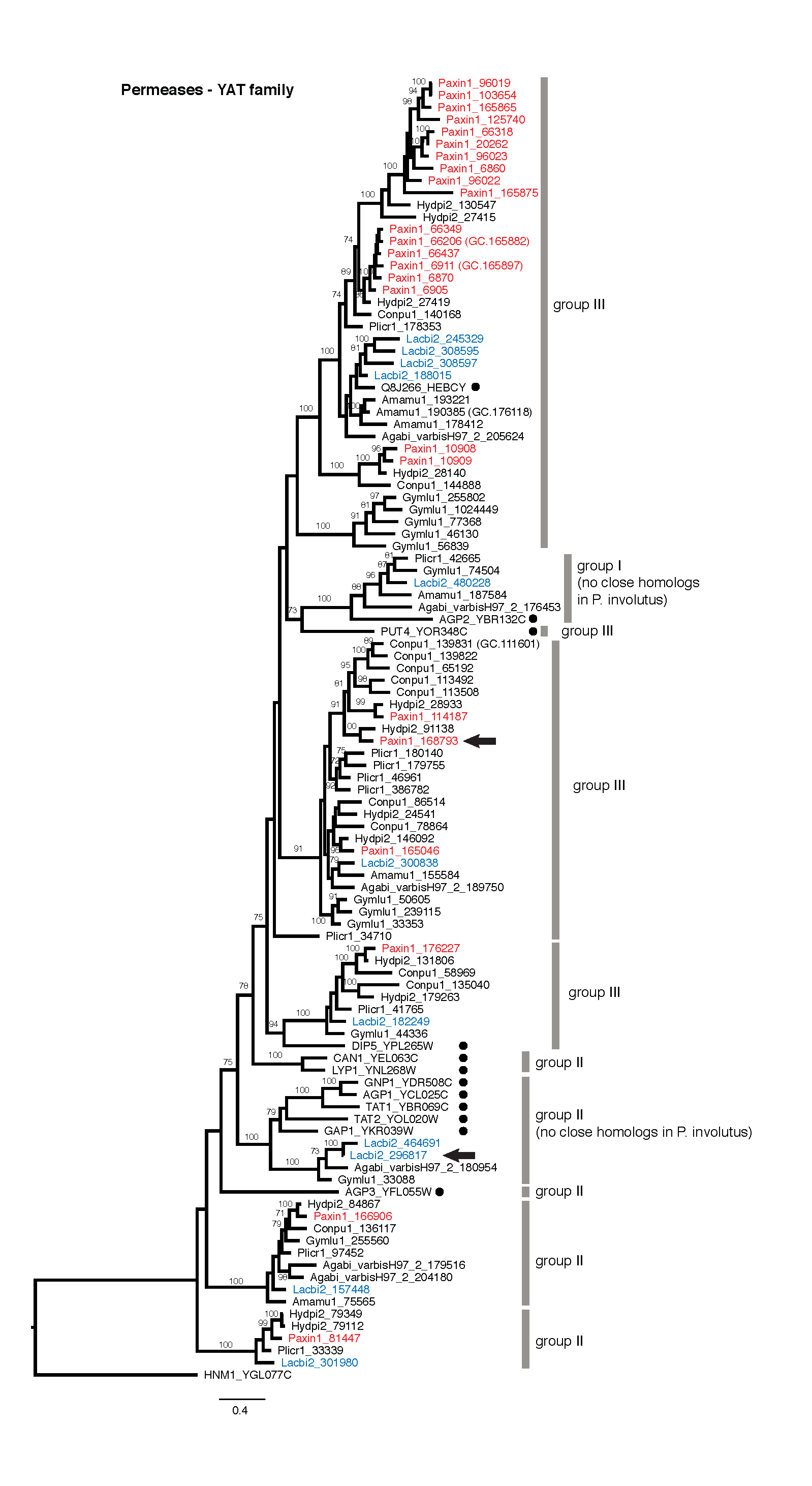
**

**
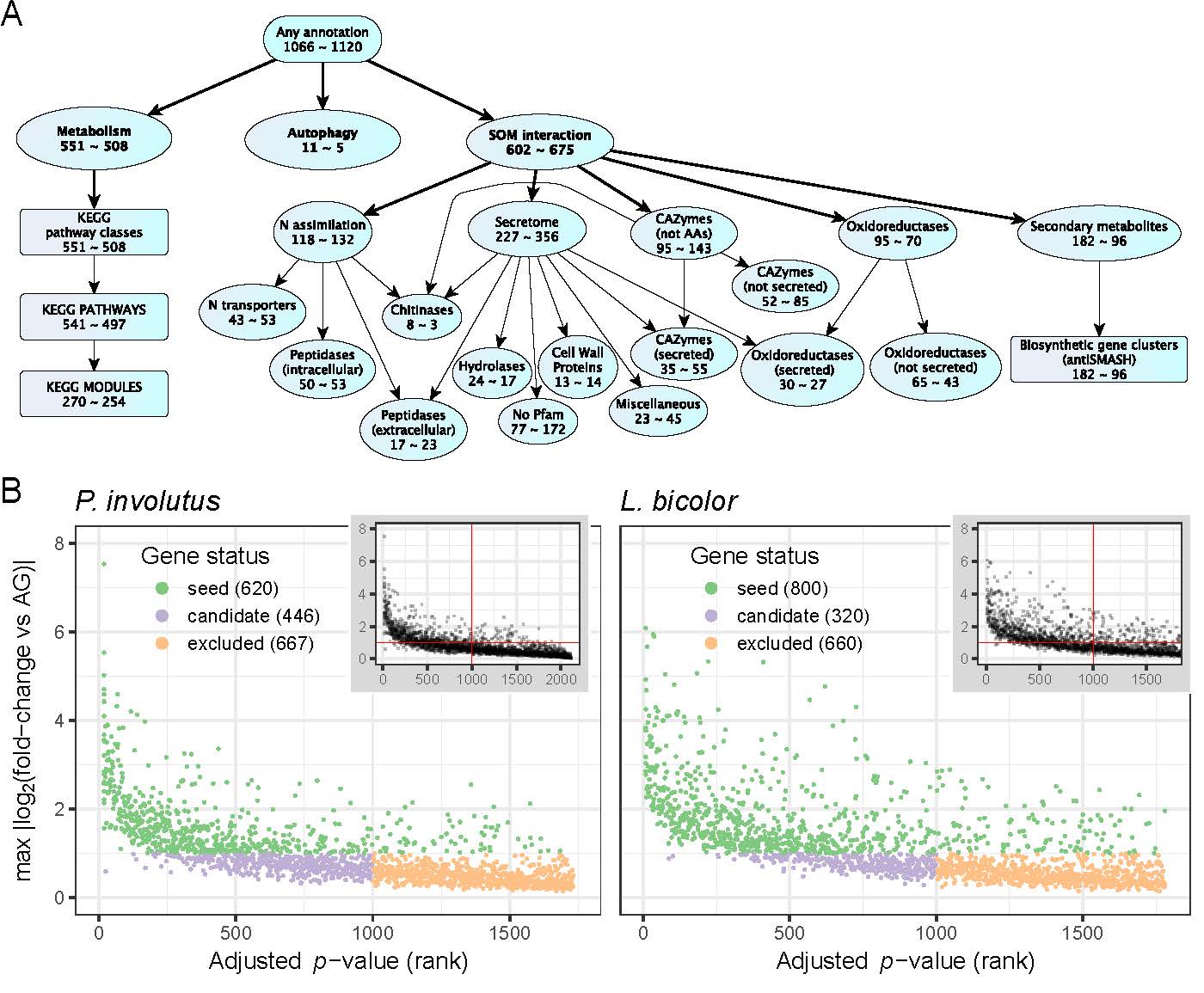
Figure S8**. Annotation scheme and selection of genes used in the FunPat co-expression analysis. (A) Annotation hierarchy. Each node represents an annotation category (ellipse) or a collection of related categories (rectangle). The further away from the root node (“Any annotation”), the more specific is the annotation category. Numbers indicate the effective number of gene models (*P. involutus*~ *L. bicolor*) in a category as input to FunPat according to the expression filtering criteria (panel B); input totals are in the root node. (B) Expression filtering criteria applied to all annotated and significant time-DEGs in *P. involutus* (1733 initial genes) and *L. bicolor* (1780 initial genes). Each dot represents one gene model. Insert shows the distribution of all annotated genes irrespective of *p*-value.

**Figure S9**. Temporal co-expression profiles and response type significance. (A) Gene set expression pattern profiles in *P. involutus* and *L. bicolor* representing a cluster of genes as identified in the FunPat co-expression analysis, grouped by response type (columns r_1_ to r_15_) and ordered (rows) according to the annotation hierarchy in Fig. S8A. The *-prefix indicates the specificity level of these 133 annotation categories associated with a gene set pattern in either fungus. Colors of the expression patterns indicate different main patterns (gene set pattern clusters) within each response type. (B) Expression profiles of the main patterns in *P. involutus* (PAI) and *L. bicolor* (LAB), with colors as in panel A and the assigned response types. Response types enriched (larger boldface font) or depleted (smaller normal font) for genes within SOM interaction (SOM) or metabolism (KEGG) annotation categories are indicated. To identify which response types are associated with a significant qualitative temporal expression pattern, the main significance criterion of interest to the current study is the enrichment for SOM-interaction genes. Most of the response types shown in Fig. 4C were significantly enriched for SOM-interaction genes in at least one species. Response type r_5_, however, was not enriched for genes from any main annotation category, but was significantly depleted for metabolism genes in both species. In this respect, r_5_ does represent a significant temporal expression profile in that the metabolic response of specific up-regulation during ND (t_2_) is a significantly narrowed response, suggesting a focused metabolism, whereas the SOM interaction is not unusual.


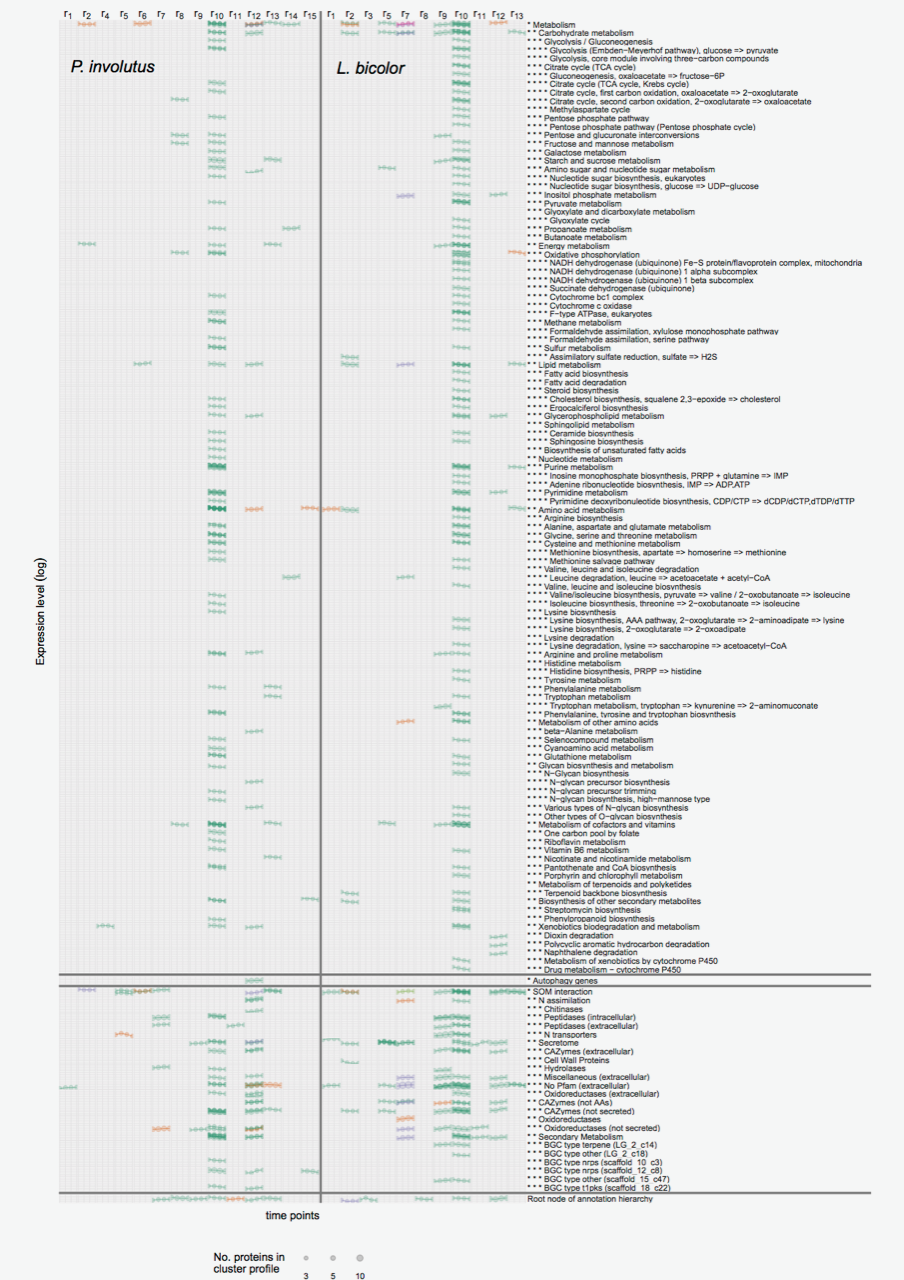
**A**

**B**

**
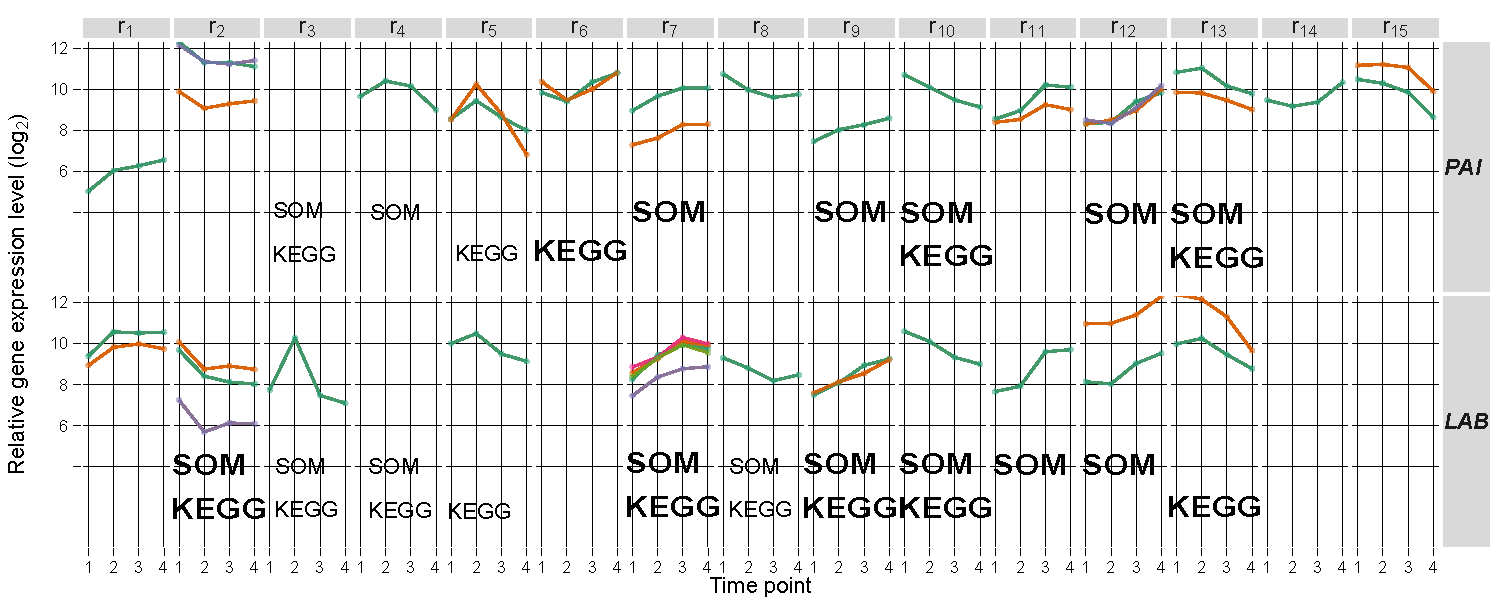
**

**Table S1.** Identification of secondary metabolite biosynthesis gene clusters in the *P. involutus* genome using antiSMASH. In total 29 clusters containing 638 proteins (72 signature proteins) were identified.

| **Gene cluster (id)** | **Type** | **Num prot^*^** | **Sign prot id^†^** | **Description** | **Annotation^‡^** |
| --- | --- | --- | --- | --- | --- |
| scaffold_10_c3 | nrps | 15 | 113133 | Siderophore synthetase | nps1 |
| scaffold_40_c4 | nrps | 29 | 82647 | Hybrid PKS-NPS | pks-nps1 (part) |
| scaffold_40_c4 | nrps |  | 82693 | Hybrid PKS-NPS | pks-nps1 (part) |
| scaffold_40_c4 | nrps |  | 82703 | Hybrid PKS-NPS | pks-nps1 (part) |
| scaffold_40_c4 | nrps |  | 82656 | Hybrid PKS-NPS | pks-nps1 (part) |
| scaffold_109_c4 | other | 3 | 17112 | AMP-dependent synthetase/ligase domain protein |  |
| scaffold_1416_c17 | other | 1 | 103648 | Nonribosomal peptide synthetase | nps2 |
| scaffold_16_c18 | other | 18 | 77684 | Quinone synthetase | invA5 |
| scaffold_16_c19 | other | 12 | 169334 | AMP-dependent synthetase/ligase domain protein |  |
| scaffold_20_c3 | other | 19 | 100025 | AMP-dependent synthetase/ligase domain protein |  |
| scaffold_20_c3 | other |  | 163299 | Alpha-aminoadipate reductase | lys2 |
| scaffold_20_c3 | other |  | 78955 | NAD-dependent epimerase/dehydratase |  |
| scaffold_20_c3 | other |  | 169671 | L-iditol 2-dehydrogenase |  |
| scaffold_4_c3 | other | 31 | 127875 | Quinone synthetase | invA4 |
| scaffold_5_c8 | other | 41 | 161353 | AMP-dependent synthetase/ligase domain protein |  |
| scaffold_12_c10,scaffold_12_c8,scaffold_12_c9 | other,nrps,other | 203 | 75867 | Hybrid PKS-NPS | pks-nps2 (part) |
| scaffold_12_c10,scaffold_12_c8,scaffold_12_c9 | other,nrps,other |  | 113799 | Hybrid PKS-NPS | pks-nps2 (part) |
| scaffold_12_c10,scaffold_12_c8,scaffold_12_c9 | other,nrps,other |  | 32164 | Hybrid PKS-NPS | pks-nps2 (part) |
| scaffold_12_c10,scaffold_12_c8,scaffold_12_c9 | other,nrps,other |  | 99020 | Hybrid PKS-NPS | pks-nps2 (part) |
| scaffold_12_c10,scaffold_12_c8,scaffold_12_c9 | other,nrps,other |  | 99027 | Hybrid PKS-NPS | pks-nps3 |
| scaffold_12_c10,scaffold_12_c8,scaffold_12_c9 | other,nrps,other |  | 11135 | AMP-dependent synthetase/ligase domain protein |  |
| scaffold_12_c10,scaffold_12_c8,scaffold_12_c9 | other,nrps,other |  | 76105 | AMP-dependent synthetase/ligase domain protein |  |
| scaffold_12_c10,scaffold_12_c8,scaffold_12_c9 | other,nrps,other |  | 168694 | AMP-dependent synthetase/ligase domain protein |  |
| scaffold_12_c10,scaffold_12_c8,scaffold_12_c9 | other,nrps,other |  | 11143 | AMP-dependent synthetase/ligase domain protein |  |
| scaffold_12_c10,scaffold_12_c8,scaffold_12_c9 | other,nrps,other |  | 99058 | AMP-dependent synthetase/ligase domain protein |  |
| scaffold_12_c10,scaffold_12_c8,scaffold_12_c9 | other,nrps,other |  | 132712 | AMP-dependent synthetase/ligase domain protein |  |
| scaffold_12_c10,scaffold_12_c8,scaffold_12_c9 | other,nrps,other |  | 168719 | AMP-dependent synthetase/ligase domain protein |  |
| scaffold_12_c10,scaffold_12_c8,scaffold_12_c9 | other,nrps,other |  | 11094 | Beta-ketoacyl synthase domain protein |  |
| scaffold_12_c10,scaffold_12_c8,scaffold_12_c9 | other,nrps,other |  | 180362 | Beta-ketoacyl synthase domain protein |  |
| scaffold_12_c10,scaffold_12_c8,scaffold_12_c9 | other,nrps,other |  | 11210 | Phosphopantetheine (PP)- and NAD binding domains protein |  |
| scaffold_12_c10,scaffold_12_c8,scaffold_12_c9 | other,nrps,other |  | 11204 | Short-chain dehydrogenases/reductase domain protein |  |
| scaffold_12_c10,scaffold_12_c8,scaffold_12_c9 | other,nrps,other |  | 11206 | Short-chain dehydrogenases/reductase domain protein |  |
| scaffold_12_c10,scaffold_12_c8,scaffold_12_c9 | other,nrps,other |  | 113899 | Short-chain dehydrogenases/reductase domain protein |  |
| scaffold_12_c10,scaffold_12_c8,scaffold_12_c9 | other,nrps,other |  | 113936 | Short-chain dehydrogenases/reductase domain protein |  |
| scaffold_12_c10,scaffold_12_c8,scaffold_12_c9 | other,nrps,other |  | 168720 | Short-chain dehydrogenases/reductase domain protein |  |
| scaffold_12_c10,scaffold_12_c8,scaffold_12_c9 | other,nrps,other |  | 168725 | Short-chain dehydrogenases/reductase domain protein |  |
| scaffold_12_c10,scaffold_12_c8,scaffold_12_c9 | other,nrps,other |  | 99026 | Poorly characterized protein |  |
| scaffold_4_c2,scaffold_4_c3 | other,other | 26 | 69019 | Quinone synthetase | invA2 |
| scaffold_4_c2,scaffold_4_c3 | other,other |  | 127833 | Quinone synthetase | invA3 |
| scaffold_4_c2,scaffold_4_c3 | other,other |  | 166727 | Aromatic amino acid aminotransferase I |  |
| scaffold_4_c2,scaffold_4_c3 | other,other |  | 8109 | Alcohol dehydrogenase zinc-binding domain-containing protein |  |
| scaffold_1654_c20 | t1pks | 1 | 103786 | Beta-ketoacyl synthase domain protein |  |
| scaffold_18_c21 | t1pks | 20 | 115494 | Beta-ketoacyl synthase domain protein | pks2 (part) |
| scaffold_18_c21 | t1pks |  | 115506 | Reducing PKS | pks2 (part) |
| scaffold_18_c21 | t1pks |  | 48358 | Phosphopantetheine (PP)-binding domain protein | pks2 (part) |
| scaffold_18_c22 | t1pks | 56 | 176643 | AMP-dependent synthetase/ligase domain protein |  |
| scaffold_18_c22 | t1pks |  | 12391 | Reducing PKS | pks3 |
| scaffold_18_c22 | t1pks |  | 12420 | Branched-chain-amino-acid transaminase |  |
| scaffold_18_c22 | t1pks |  | 99888 | Branched-chain-amino-acid transaminase |  |
| scaffold_18_c22 | t1pks |  | 169521 | Branched-chain-amino-acid transaminase |  |
| scaffold_18_c22 | t1pks |  | 78469 | Branched-chain-amino-acid transaminase |  |
| scaffold_8_c14 | t1pks | 44 | 9490 | Nonreducing PKS | pks1 |
| scaffold_8_c14 | t1pks |  | 167559 | Short-chain dehydrogenases/reductase domain protein |  |
| scaffold_11_c7 | terpene | 20 | 11037 | AMP-dependent synthetase/ligase domain protein |  |
| scaffold_11_c7 | terpene |  | 168647 | Lanosterol synthase |  |
| scaffold_13_c14 | terpene | 9 | 180528 | Terpene synthase |  |
| scaffold_2_c1 | terpene | 8 | 165670 | Alanine-glyoxylate transaminase |  |
| scaffold_2_c1 | terpene |  | 165668 | Lanosterol synthase |  |
| scaffold_2_c2 | terpene | 10 | 160473 | Squalene synthase |  |
| scaffold_21_c4 | terpene | 8 | 12806 | Terpene synthase |  |
| scaffold_26_c7 | terpene | 10 | 170150 | Lanosterol synthase |  |
| scaffold_351_c11 | terpene | 5 | 18633 | Terpene synthase |  |
| scaffold_4_c1 | terpene | 19 | 166668 | Poorly characterized protein |  |
| scaffold_4_c1 | terpene |  | 166672 | Quinone synthetase | invA1 |
| scaffold_4_c1 | terpene |  | 69028 | Quinone synthetase | invA6 |
| scaffold_41_c5 | terpene | 7 | 171189 | Squalene synthase |  |
| scaffold_7_c10 | terpene | 23 | 167347 | AMP-dependent synthetase/ligase domain protein |  |
| scaffold_7_c10 | terpene |  | 97557 | AMP-dependent synthetase/ligase domain protein |  |
| scaffold_7_c10 | terpene |  | 9124 | NAD binding domain protein |  |
| scaffold_7_c10 | terpene |  | 167348 | Terpene synthase |  |
| scaffold_8_c15 | terpene | 11 | 73166 | Poorly characterized protein |  |
| scaffold_82_c19 | terpene | 11 | 86018 | Poorly characterized protein |  |

^*^ Number of proteins in the cluster predicted by the antiSMASH algorithm (30).

^†^ Proteins or protein domains identified by antiSMASH that are characteristic for the predicted gene clusters.

^‡^ Manually annotated genes in Shah et al. (27).

**Table S2**. Identification of secondary metabolite biosynthesis gene clusters in the *L. bicolor* genome using antiSMASH. In total 20 clusters containing 576 proteins (26 signature proteins) were identified.

| **Gene cluster (id)** | **Type** | **Num prot^*^** | **Sign prot id^†^** | **Description** | **Annotation^‡^** |
| --- | --- | --- | --- | --- | --- |
| LG_1_c5 | indole | 12 | 479299 | Tryptophan dimethylallyltransferase domain protein |  |
| LG_1_c8 | other | 25 | 574577 | AMP-dependent synthetase and ligase domain protein |  |
| LG_1_c8 | other |  | 640838 | AMP-dependent synthetase and ligase domain protein |  |
| LG_2_c18 | other | 19 | 184376 | Alpha-aminoadipate reductase | lys2 |
| LG_5_c33 | other | 19 | 333039 | AMP-dependent synthetase and ligase domain protein |  |
| scaffold_15_c47 | other | 211 | 253196 | AMP-dependent synthetase and ligase domain protein |  |
| scaffold_15_c47 | other |  | 452259 | 3-oxoacyl-[acyl-carrier protein] reductase |  |
| scaffold_15_c47 | other |  | 617118 | AMP-dependent synthetase and ligase domain protein |  |
| scaffold_15_c47 | other |  | 616666 | AMP-dependent synthetase and ligase domain protein |  |
| LG_3_c21 | siderophore | 16 | 442046 | Siderophore biosynthesis domain protein |  |
| LG_3_c21 | siderophore |  | 587012 | Siderophore biosynthesis domain protein |  |
| LG_10_c10 | t1pks | 56 | 475034 | Nonreducing PKS | pks1 |
| LG_4_c31 | t1pks | 60 | 244979 | Nonreducing PKS | pks |
| LG_10_c12 | terpene | 9 | 394925 | Terpene synthase |  |
| LG_10_c9 | terpene | 9 | 707333 | Squalene synthase |  |
| LG_2_c14 | terpene | 57 | 683499 | Terpene synthase |  |
| LG_3_c24 | terpene | 8 | 686603 | Squalene synthase |  |
| LG_4_c28 | terpene | 13 | 182495 | Squalene synthase |  |
| LG_4_c30 | terpene | 12 | 443788 | Terpene synthase metal binding domain protein |  |
| LG_6_c34 | terpene | 12 | 484390 | Terpene synthase |  |
| LG_8_c37 | terpene | 10 | 393120 | Terpene synthase metal binding domain protein |  |
| scaffold_11_c41 | terpene | 7 | 312850 | Terpene synthase |  |
| scaffold_11_c42 | terpene | 10 | 611606 | Poorly characterized |  |
| scaffold_14_c46 | terpene | 10 | 653994 | Poorly characterized |  |

^*^ Number of proteins in the cluster predicted by the antiSMASH algorithm (30).

^†^ Proteins or protein domains identified by antiSMASH that are characteristic for the predicted gene clusters.

^‡^ Manually annotated genes in Shah et al. (27).

**Table S3.** Vibrational frequencies (cm^-1^) from FTIR spectra and assignments for the soil organic matter extract.

| **Position**  **(cm^-1^)** | **Functional groups** | **Compound** | **Attributed reaction** |
| --- | --- | --- | --- |
| 1720 | Carboxyl acid C=O stretch | Oxidized products | Carboxyl formation |
| 1628 | Ketone C=O stretch | Polyconjugated systems | Aromatic accumulation |
| 1510 | Aromatic skeletal vibration | Lignin | Lignin accumulation |
| 1420 | C-H, O-H bending | Alkanes, phenols | Polysaccharides decomposition |
| 1380 | N-H | Ammonium | NH_4_^+^ assimilation |
| 1215 | C-C, C-O, C=O | Lignin | Carboxyl formation |
| 1020 | C-O | Glucose | C_gluc_ assimilation |

**Table S4.** Organic N composition of the soil organic matter extract incubated with *P. involutus* and *L. bicolor.* Relative abundance of different types of organic N (mean ± standard error, *n* = 3) calculated based on the relative area of the deconvoluted area of N K-edge peak spectra (see Fig. S5). Types of N: non-peptide C=N as in aliphatic imines and/or in aromatic pyridines, pyrazines, pyrimidines (399.2 eV); (b) nitriles and/or aromatic systems such as purine, pyrazole and/or imidazole (401.2 eV); (c) amide/amine N (401.5 eV) and (d) N-heterocyclic aromatics such as pyrrole (403 eV).

| **N-type** | **Energy**  **(eV)** | **Initial SOM extract** | ***Paxillus involutus*** | | | | ***Laccaria bicolor*** | | | |
| --- | --- | --- | --- | --- | --- | --- | --- | --- | --- | --- |
|  |  | t_0_ | t_1_ | t_2_ | t_3_ | t_4_ | t_1_ | t_2_ | t_3_ | t_4_ |
| Heterocyclic-N | 399.2 | 4.7 (0.3) | 3.1 (0.7) | 1.2 (0.4) | 4.7 (0.6) | 5.4 (0.1) | 3.2 (0.3) | 4.4 (0.1) | 7.2 (0.4) | 7.3 (0.1) |
| Nitrile N | 400.2 | 6.3 (0.1) | 6.7 (0.6) | 6.7 (0.6) | 8.2 (0.7) | 6.1 (0.4) | 7.9 (0.4) | 7.4 (0.3) | 7.2 (0.3) | 6.5 (0.6) |
| Amide N | 401.5 | 58.5 (0.9) | 58.7 (0.9) | 58.7 (0.9) | 52.9 (1.1) | 53.6 (0.5) | 58.5 (0.5) | 55.8 (0.3) | 52.6 (1.4) | 51.9 (0.4) |
| Pyrrole N | 403 | 30.5 (0.7) | 31.5 (0.4) | 31.5 (0.4) | 34.1 (0.4) | 34.9 (0.1) | 30.4 (0.6) | 32.4 (0.1) | 33.1 (0.7) | 34.2 (0.4) |

**Table S5.** Statistics of RNA sequencing (RNA-Seq). Shown are the number of raw reads, number of removed rRNA reads and the number of reads that were mapped to the transcriptome.

| **Sample** | **Raw reads** | **rRNA- reads**  **(removed)** | **rRNA**  **(% of raw reads)** | **Mapped**  **reads** | **Mapped (%)** |
| --- | --- | --- | --- | --- | --- |
| LAB T1-Rep1 | 14591141 | 173435 | 1.2 | 12028272 | 82.4 |
| LAB T1-Rep2 | 16136908 | 371339 | 2.3 | 13126905 | 81.3 |
| LAB T1-Rep3 | 14492448 | 465492 | 3.2 | 11659746 | 80.5 |
| LAB T2-Rep1 | 16142141 | 197575 | 1.2 | 13100757 | 81.2 |
| LAB T2-Rep2 | 15549013 | 196226 | 1.3 | 12604681 | 81.1 |
| LAB T2-Rep3 | 15391443 | 217077 | 1.4 | 12465756 | 81 |
| LAB T3-Rep1 | 14419300 | 187941 | 1.3 | 11583145 | 80.3 |
| LAB T3-Rep2 | 15495007 | 506569 | 3.3 | 12221411 | 78.9 |
| LAB T3-Rep3 | 16702089 | 936969 | 5.6 | 12762745 | 76.4 |
| LAB T4-Rep1 | 17676086 | 514785 | 2.9 | 13772019 | 77.9 |
| LAB T4-Rep2 | 14752035 | 234964 | 1.6 | 11675542 | 79.1 |
| LAB T4-Rep3 | 18692387 | 368232 | 2 | 14644851 | 78.3 |
| PAI T1-Rep1 | 16424087 | 134161 | 0.8 | 12764443 | 77.7 |
| PAI T1-Rep2 | 14694572 | 140634 | 1 | 11421834 | 77.7 |
| PAI T1-Rep3 | 19631161 | 295680 | 1.5 | 14951187 | 76.2 |
| PAI T2-Rep1 | 14005536 | 236452 | 1.7 | 10734669 | 76.6 |
| PAI T2-Rep2 | 14501431 | 213644 | 1.5 | 11090001 | 76.5 |
| PAI T2-Rep3 | 15964533 | 258648 | 1.6 | 12176917 | 76.3 |
| PAI T3-Rep1 | 15196392 | 246217 | 1.6 | 11275775 | 74.2 |
| PAI T3-Rep2 | 15317649 | 931742 | 6.1 | 10878325 | 71 |
| PAI T3-Rep3 | 16126090 | 308635 | 1.9 | 11983108 | 74.3 |
| PAI T4-Rep1 | 15190257 | 277152 | 1.8 | 11242024 | 74 |
| PAI T4-Rep2 | 16136474 | 257752 | 1.6 | 11974081 | 74.2 |
| PAI T4-Rep3 | 15740766 | 281262 | 1.8 | 11564979 | 73.5 |

**Table S6.** Differentially expressed transcripts in *P. involutus* and *L. bicolor* during growth on organic matter extract. Shown is the number of transcripts that were differentially regulated in at least one time point (t_1_ to t_4_ cf. Fig. 1, main text).

| **Gene categories** ^*^ | **Genome**^†^ | **Transcr.‡** | **Cover** |  | **Regulated**^¶^ | | |  |  | |  | |  | |
| --- | --- | --- | --- | --- | --- | --- | --- | --- | --- | --- | --- | --- | --- | --- |
|  |  |  | **(%)**^§^ | t1 (high) | | t2 (high) | t3 (high) | | | t4 (high) | | Sum | | (%) |
| ***P. involutus*** |  |  |  |  | |  |  | | |  | |  | |  |
| Proteins | 17 968 | 12 103 | 67.4 | 2 860 | | 1 289 | 888 | | | 2 742 | | 7 779 | | 64.3 |
| KOG | 8 310 | 7 222 | 86.9 | 2 167 | | 885 | 521 | | | 1 586 | | 5 159 | | 71.4 |
| Cellular processes and signaling (KOG) | 2 536 | 2 217 | 87.4 | 575 | | 323 | 181 | | | 511 | | 1 590 | | 71.7 |
| Information storage and processing (KOG) | 1 881 | 1 584 | 84.2 | 584 | | 156 | 78 | | | 287 | | 1 105 | | 69.8 |
| Metabolism (KOG) | 2 310 | 2 128 | 92.1 | 687 | | 272 | 187 | | | 498 | | 1 644 | | 77.3 |
| Poorly characterized (KOG) | 2 084 | 1 735 | 83.3 | 449 | | 192 | 110 | | | 386 | | 1 137 | | 65.5 |
| Undefined (KOG) | 15 | 12 | 80 | 3 | | 1 | 0 | | | 3 | | 7 | | 58.3 |
| Metabolism (KEGG) | 1 039 | 1 030 | 99.1 | 453 | | 136 | 50 | | | 195 | | 834 | | 81 |
| Secondary metabolism (AntiSMASH) | 72 | 72 | 100 | 23 | | 13 | 8 | | | 13 | | 57 | | 79.2 |
| SMBG | 11 | 11 | 100 | 4 | | 3 | 0 | | | 4 | | 11 | | 100 |
| Peptidases (secreted) | 41 | 40 | 97.6 | 9 | | 9 | 7 | | | 7 | | 32 | | 80 |
| Peptidases (non-secreted) | 177 | 175 | 98.9 | 58 | | 22 | 13 | | | 43 | | 136 | | 77.7 |
| N-transporters | 72 | 71 | 98.6 | 12 | | 17 | 15 | | | 16 | | 60 | | 84.5 |
| CAZymes | 261 | 249 | 95.4 | 72 | | 24 | 15 | | | 62 | | 173 | | 69.5 |
| Oxidases (AAs) | 33 | 33 | 100 | 11 | | 2 | 4 | | | 11 | | 28 | | 84.8 |
| Secreted (classical) | 640 | 506 | 79.1 | 117 | | 51 | 44 | | | 140 | | 352 | | 69.6 |
| Secreted (non-classical) | 227 | 126 | 55.5 | 24 | | 13 | 5 | | | 19 | | 61 | | 48.4 |
| ATG | 23 | 23 | 100 | 0 | | 8 | 1 | | | 12 | | 21 | | 91.3 |
| ***L. bicolor*** |  |  |  |  | |  |  | | |  | |  | |  |
| Proteins | 23 130 | 14 472 | 62.6 | 3 406 | | 1 548 | 1 092 | | | 2 873 | | 8 919 | | 61.6 |
| KOG | 8 357 | 7 096 | 84.9 | 2350 | | 821 | 532 | | | 1 380 | | 5 083 | | 71.6 |
| Cellular processes and signaling (KOG) | 2 858 | 2 433 | 85.1 | 709 | | 312 | 200 | | | 517 | | 1 738 | | 71.4 |
| Information storage and processing (KOG) | 2 088 | 1 670 | 80 | 610 | | 131 | 116 | | | 342 | | 1 199 | | 71.8 |
| Metabolism (KOG) | 2 223 | 2 008 | 90.3 | 735 | | 250 | 160 | | | 336 | | 1 481 | | 73.8 |
| Poorly characterized (KOG) | 1 822 | 1 557 | 85.5 | 453 | | 195 | 110 | | | 303 | | 1 061 | | 68.1 |
| Undefined (KOG) | 25 | 11 | 44 | 0 | | 1 | 2 | | | 5 | | 8 | | 72.7 |
| Metabolism (KEGG) | 997 | 956 | 95.9 | 453 | | 120 | 65 | | | 130 | | 768 | | 80.3 |
| Secondary metabolism (AntiSMASH) | 24 | 23 | 95.8 | 5 | | 0 | 0 | | | 10 | | 15 | | 65.2 |
| SMBG | 3 | 3 | 100 | 1 | | 0 | 0 | | | 2 | | 3 | | 100 |
| Peptidases (secreted) | 47 | 45 | 95.7 | 11 | | 4 | 4 | | | 16 | | 35 | | 77.8 |
| Peptidases (non-secreted) | 206 | 199 | 96.6 | 74 | | 29 | 13 | | | 30 | | 146 | | 73.4 |
| N-transporters | 115 | 114 | 99.1 | 31 | | 19 | 13 | | | 24 | | 87 | | 76.3 |
| CAZymes | 321 | 285 | 88.8 | 64 | | 53 | 30 | | | 61 | | 208 | | 73 |
| Oxidases (AAs) | 55 | 44 | 80 | 7 | | 12 | 5 | | | 4 | | 28 | | 63.6 |
| Secreted (classical) | 1 098 | 791 | 72 | 162 | | 124 | 68 | | | 172 | | 526 | | 66.5 |
| Secreted (non-classical) | 332 | 138 | 41.6 | 18 | | 19 | 9 | | | 24 | | 70 | | 50.7 |
| ATG | 23 | 23 | 100 | 8 | | 3 | 3 | | | 3 | | 17 | | 73.9 |

^*^  “KOG” are gene models assigned to Eukaryotic Orthologous Groups (KOGs); “KEGG” are gene models assigned to metabolic pathways in the KEGG database; “Secondary metabolites” are signature genes identified using the antiSMASH tool (Tables S1 and S2); “SMBG” refer to manually identified genes presumably involved in the synthesis of secondary metabolites (27); “Peptidases” are gene models of peptidases based on search in the MEROPS database (26); “CAZymes” are gene models of carbohydrate-modifying enzymes; “AAs” are gene models encoding auxiliary redox activities/enzymes; ”Secreted” is the number of gene models that were predicted to contain either a classical or non-classical secretion signal (cf. Fig. S1); “ATG” are genes encoding autophagy related proteins.

^†^  The number of predicted gene models as by the Joint Genome Institute (JGI) MycoCosm portal: *P. involutus* <http://genome.jgi-psf.org/Paxin1/Paxin1.home.html>; *L. bicolor* (<http://genome.jgi-psf.org/Lacbi2/Lacbi2.home.html> (13).

^‡^ The number of predicted gene models that were found to be transcribed in the present experiments.

^§^ The proportion of predicted gene models that were transcribed in the present experiments.

^¶^ “t_1_ (high)… t_4_ (high)” is the number of genes that were most highly expressed at t_1_, t_2,_ t_3_ and t_4,_ respectively (*p*_adj_<0.01, *n*=3); “Sum” is the total number of time-regulated genes; “%” indicates the proportion of transcribed gene that were time-regulated.

**Table S7.** Functional features of the most highly up-regulated and expressed genes at the onset of SOM oxidation in *P. involutus*. Of the 20% most highly expressed genes at t_2_, 62 genes were up-regulated more than twofold in the pairwise comparisons of samples from t_2_ and t_1_ (*n*=3, *p*_adj_ <0.01). Based on protein homology searches, 50 of these genes were annotated to various functional categories. The category “Poorly characterized” includes proteins with protein (PFAM) domains that could not be annotated to specific functions. The remaining highly expressed and up-regulated genes lacked PFAM domains (12 genes). Ft2, Ft3, Ft4 are the pairwise fold changes of t_2_, t_3_ and t_4_ vs. t_1_.

| **Protein Id** | **Functional category** | **Description** | **Ft2** | **Ft3** | **Ft4** |
| --- | --- | --- | --- | --- | --- |
| **167454** ^*,†^ | N-metabolism | DAHP synthetase (3-deoxy-7-phosphoheptulonate synthase) (EC 2.5.1.54) (PF00793) | 2.47 | 0.63 | 0.23 |
| 104146 | N-metabolism | Amidase (EC 3.5.1.4) (PF01425) | 2.44 | 1.87 | 0.50 |
| 71407 | N-metabolism | Amidohydrolase-related protein (non-secreted) (PF13147) | 2.14 | 0.97 | 0.61 |
| **69875** | N-metabolism | Cysteine aminopeptidase (C01.085 (bleomycin)) (LAP3) (**PF03051**) ^‡^ | 4.85 | 2.18 | 1.08 |
| **105760** | N-assimilation | A01 Peptidase **(PF00026**) (**secreted**) ^§^ | 3.85 | 1.80 | 0.68 |
| 128888 | N-assimilation | Peptidase (G01.002) **(secreted**) (PF01828) | 2.29 | 1.35 | 0.78 |
| **15976** | N-assimilation | Ammonium permease (AMT family) (PF00909) | 3.70 | 3.06 | 0.73 |
| **76416** | N-assimilation | Nitrate transporter (NNP family) (**PF07690**) | 5.93 | 1.23 | 0.16 |
| **164294** | N-assimilation | Oligopeptide transporter (OPT family) (**PF03169**) | 4.96 | 9.52 | 5.37 |
| 74571 | N-assimilation | Oligopeptide transporter (OPT family) (**PF03169**) | 2.39 | 2.44 | 2.91 |
| **172505** | N-assimilation | Urea and polyamine transporter PiDur3 (SSS family) (PF00474) | 2.39 | 1.23 | 0.46 |
| **168793** | N-assimilation | Amino acid permease (YAT family) (**PF00324**) | 2.22 | 3.13 | 2.87 |
| 136965 | C-metabolism  (Aromatic-C) | Dioxygenase (DOPA) (PF08883) | 2.35 | 3.14 | 3.16 |
| 165694 | C-metabolism  (Aromatic-C) | Salicylate hydroxylase (EC 1.14.13.1) **(PF01494**) | 2.40 | 0.61 | 0.21 |
| 166108 | Iron metabolism  (Miscellaneous) | Iron sulfur cluster assembly protein (PF01592) | 2.58 | 2.15 | 2.35 |
| 168719 | Secondary metabolism  (Miscellaneous) | NRPS domain protein (PF00501,PF07993) | 2.73 | 3.22 | 0.50 |
| 170472 | Oxidoreductases | Cytochromes P450 (**PF00067**) | 2.09 | 2.30 | 2.53 |
| 170639 | Signal transduction | RAS small GTPase (PF00071) | 3.14 | 3.00 | 1.80 |
| 163479 | Signal transduction | RhoGEF domain, CNH domain (PF00169, PF00780) | 2.46 | 3.19 | 5.91 |
| 17458 | Signal transduction | Serine/threonine protein kinase (putative) (**PF07714**) | 2.33 | 3.20 | 7.45 |
| *80503* ^e^ | Cellular transport | Cation ATPase (**PF00122**,**PF00689**,**PF00690**,**PF00702**) | 2.64 | 4.28 | 2.35 |
| 168761 | Cellular transport | Major Facilitator Superfamily transporter (**PF07690**) | 2.21 | 1.04 | 0.59 |
| 168887 | Cellular transport | Major Facilitator Superfamily transporter (**PF07690**) | 2.07 | 8.11 | 7.24 |
| 173058 | Cellular transport | Multi Antimicrobial Extrusion (MATE) family protein (PF01554) | 7.32 | 8.16 | 1.01 |
| 162541 | Intracellular trafficking | Autophagy-related protein 2 (PF09333,PF12624,PF13329) | 2.18 | 2.05 | 1.95 |
| 159774 | Intracellular trafficking | Vacuolar protein sorting-associated protein 13 (PF06650,PF09333,PF12624) | 2.09 | 2.36 | 1.89 |
| 169793 | Cytoskeleton | Beta-tubulin | 2.00 | 1.24 | 1.08 |
| 164151 | Protein turnover | Ubiquitin-conjugating enzyme, E2 (**PF00179**) | 2.44 | 2.32 | 1.65 |
| 109974 | Transcription | Fungal Zn(2)-Cys(6) domain protein (**PF00172**) | 2.70 | 1.63 | 1.71 |
| 166547 | Poorly characterized | 14-3-3 proteins domain protein (PF00244) | 2.27 | 1.47 | 1.44 |
| 100273 | Poorly characterized | AIG1 family domain protein (PF04548) | 3.26 | 1.90 | 1.11 |
| 170907 | Poorly characterized | DUF2235 **(PF09994**) | 2.10 | 2.46 | 1.60 |
| 169455 | Poorly caharcterized | DUF3602 (PF12223) | 2.17 | 2.47 | 1.17 |
| 7975 | Poorly characterized | DUF4243 **(PF14027**) | 3.36 | 1.72 | 0.29 |
| 165331 | Poorly characterized | DUF89 (PF01937) | 3.06 | 3.14 | 1.72 |
| 77690 | Poorly characterized | DUF914 (PF06027) | 2.16 | 1.55 | 0.62 |
| 167498 | Poorly characterized | DUF914 (PF06027) | 2.40 | 1.65 | 0.97 |
| 171167 | Poorly characterized | Dynamin family protein (GTPase ) (**PF00350**,PF01031,PF02212) | 2.03 | 3.67 | 1.46 |
| 75823 | Poorly characterized | Class II Aldolase and Adducin N-terminal domain protein (PF00596) | 2.16 | 1.53 | 0.90 |
| 73504 | Poorly characterized | Est1 DNA/RNA binding domain, PIN 4 domain (PF10373,PF13638) | 2.67 | 2.75 | 2.03 |
| 162160 | Poorly characterized | Fasciclin (FAS1) domain protein (PF02469) **(secreted**) | 2.89 | 2.64 | 1.42 |
| 106065 | Poorly characterized | F-box-like domain (**PF12937**) | 2.26 | 4.82 | 5.85 |
| 25650 | Poorly characterized | Metallo-beta-lactamase protein fold domain (PF00753) | 2.50 | 3.57 | 4.30 |
| 165369 | Poorly characterized | PB1 domain protein (PF00564) | 3.50 | 3.17 | 1.85 |
| 169359 | Poorly characterized | RNA recognition motif, RNP-1domain protein (PF00076) | 2.04 | 0.54 | 0.42 |
| 169306 | Poorly characterized | S1 and P1 nucleases domain protein (PF02265) (**secreted**) | 2.39 | 6.15 | 5.05 |
| 115601 | Poorly characterized | SDR domain protein (**PF00106**) | 2.23 | 1.16 | 0.91 |
| 167429 | Poorly characterized | SDR domain protein (**PF00106**) (**secreted)** | 2.86 | 2.18 | 0.93 |
| 129191 | Poorly characterized | Transient receptor potential (TRP) ion channel (PF06011) | 2.08 | 1.66 | 1.29 |
| 154238 | Poorly characterized | Zinc finger (Znf) domain protein (PF13465) | 2.04 | 4.21 | 2.65 |

^*^ Proteins in bold letters are displayed in Fig. 3.

^†^ Underlined are *P. involutus* proteins that have a 1:1 orthologue identified in the *L. bicolor* genome.

^‡^ PFAM domains in bold letters represent 12 domains that were identified among the most highly up-regulated and expressed transcripts at the onset of SOM oxidation in both *P. involutus* and *L. bicolor*.

^§^ Predicted secretion signal.

^¶^ Protein id in italics indicates a 1:1 orthologue that was found among the up-regulated and expressed transcripts at the onset of SOM oxidation in both *P. involutus* and *L. bicolor* (cf. Table S8).

**Table S8.** Functional features of the most highly up-regulated and expressed genes at the onset of SOM oxidation in *L. bicolor*. Of the 20% most highly expressed genes at t_2_, 225 genes were up-regulated more than twofold in the pairwise comparisons of samples from t_2_ and t_1_ (*n*=3, *p*_adj_ <0.01). Based on protein homology searches, 129 of these genes were annotated to various functional categories. The category “Poorly characterized” includes proteins with protein (PFAM) domains that could not be annotated to specific functions. The remaining highly expressed and up-regulated genes lacked PFAM domains (96 genes). Ft2, Ft3, Ft4 are the pairwise fold changes of t_2_, t_3_ and t_4_ vs. t_1_.

| **Prot id** | **Functional category** | **Description** | **Ft2** | **Ft3** | **Ft4** |
| --- | --- | --- | --- | --- | --- |
| **188701** ^*,†^ | N-metabolism | 1-pyrroline dehydrogenase (PUT2) (EC 1.2.1.88) | 2.95 | 1.35 | 1.12 |
| 307566 | N-metabolism | Aldehyde dehydrogenase (NAD+) (EC 1.2.1.3) | 2.21 | 0.41 | 0.09 |
| 475428 | N-metabolism | Ethanolamine utilisation protein EutQ (putative) | 3.43 | 1.70 | 1.71 |
| **182694** | N-metabolism | Glutamate dehydrogenase ( EC 1.4.1.2) | 3.21 | 2.38 | 1.71 |
| 254229 | N-metabolism | Glutamate decarboxylase (EC 4.1.1.15) | 3.42 | 1.72 | 0.87 |
| **144077** | N-assimilation | A01 Peptidase **(PF00026**) ^‡^ (**secreted**) ^§^ | 3.83 | 0.94 | 0.81 |
| **635298** | N-assimilation | Cysteine aminopeptidase (C01.085, bleomycin) (LAP3) (**PF03051**) | 27.99 | 12.04 | 2.80 |
| 669910 | N-assimilation | Peptidase (C02, calpain) (PF00648,PF01067,PF04212) | 2.27 | 2.09 | 1.43 |
| 668546 | N-assimilation | Peptidase (C14, caspase catalytic) (PF00656) | 2.72 | 1.75 | 1.19 |
| 297571 | N-assimilation | The anion:cation symporter family (ACS) (**PF07690**) | 4.59 | 18.34 | 4.81 |
| 312917 | N-assimilation | The drug:H+ antiporter-1 family (DHA1) (**PF07690**) | 7.39 | 6.67 | 3.29 |
| **242631** | N-assimilation | Oligopeptide transporter (OPT family) (**PF03169**) | 3.43 | 5.68 | 4.22 |
| 301981 | N-assimilation | The peptide transporter family (PTR) (PF00854) | 2.75 | 36.58 | 59.58 |
| 182249 | N-assimilation | Amino acid permease (YAT family) (**PF00324**) | 2.39 | 1.49 | 0.88 |
| **296817** | N-assimilation | Amino acid permease (YAT family) (**PF00324**) | 5.48 | 6.08 | 1.68 |
| 692663 | C-metabolism  (Lipid metabolism) | Lipoxygenase (EC 1.13.11.-) | 2.50 | 5.47 | 3.63 |
| 694218 | C-metabolism  (Lipid metabolism) | Phosphatidylserine decarboxylase (EC 4.1.1.65) | 2.07 | 2.13 | 2.74 |
| 702542 | C-metabolism  (Lipid metabolism) | Phosphatidylserine decarboxylase (EC 4.1.1.65) | 3.31 | 6.20 | 7.14 |
| 697540 | C-metabolism  (Lipid metabolism) | Phospholipase D (EC 3.1.4.4) | 2.81 | 1.61 | 1.02 |
| 669666 | Iron metabolism  (Miscellaneous) | Ferric reductase like transmembrane protein (PF01794,PF08022,PF08030) | 2.00 | 1.11 | 0.82 |
| **253895** | CAZymes | Chitin deacetylase (CE4) (**secreted**) | 2.63 | 2.78 | 2.52 |
| **293318** | CAZymes | Chitin deacetylase (CE4) (**secreted**) | 3.89 | 7.16 | 5.70 |
| **307596** | CAZymes | Chitin deacetylase (CE4) (**secreted**) | 12.53 | 2.17 | 0.53 |
| **308312** | CAZymes | Chitin deacetylase (CE4) (**secreted**) | 4.39 | 16.39 | 25.66 |
| **245379** | CAZymes | Pectin methylesterase (CE8) (**secreted**) | 2.93 | 3.89 | 3.64 |
| **247310** | CAZymes | Amylase (GH13_1) (**secreted**) | 3.01 | 14.61 | 50.79 |
| 182606 | CAZymes | GH16 (**secreted**) | 2.26 | 0.57 | 0.43 |
| 379212 | CAZymes | GH16 | 2.68 | 1.03 | 1.10 |
| 187709 | CAZymes | GH16 (**secreted**) | 4.22 | 11.21 | 16.92 |
| 296997 | CAZymes | GH17 | 4.57 | 3.22 | 4.66 |
| 236861 | CAZymes | Chitinase (GH18) | 11.85 | 6.13 | 4.27 |
| 185397 | CAZymes | Chitinase (GH18) | 7.85 | 8.75 | 8.19 |
| **313808** | CAZymes | Lysozyme/Chitinase (GH23) (**secreted**) | 2.18 | 2.50 | 2.80 |
| **239749** | CAZymes | Lysozyme (GH24) (**secreted**) | 4.29 | 3.10 | 1.27 |
| **245054** | CAZymes | A-A trehalase (GH37) (**secreted**) | 3.08 | 5.27 | 1.06 |
| 294893 | CAZymes | GH5_30 (**secreted**) | 2.03 | 0.92 | 0.42 |
| 185358 | CAZymes | GH5_9 (**secreted**) | 2.15 | 2.14 | 1.81 |
| 234059 | CAZymes | GH79 (**secreted**) | 11.90 | 1.29 | 1.16 |
| 295325 | CAZymes | GH79 (**secreted**) | 2.90 | 0.89 | 0.73 |
| 295469 | CAZymes | GH79 (**secreted**) | 6.16 | 3.25 | 1.85 |
| **313935** | Oxidoreductases | CRO-1, Copper radical oxidase (AA5_1) (**secreted**) | 4.28 | 0.75 | 0.24 |
| 291185 | Oxidoreductases | Cytochromes P450 (**PF00067**) | 3.78 | 4.21 | 4.89 |
| 670590 | Oxidoreductases | Cytochromes P450 (**PF00067**) | 2.73 | 12.39 | 9.56 |
| 671405 | Oxidoreductases | Cytochromes P450 (**PF00067**) | 2.80 | 3.14 | 2.56 |
| 318937 | Oxidoreductases | FAD binding domain (PF01565) | 6.41 | 55.22 | 53.08 |
| 482529 | Oxidoreductases | Flavin-containing monooxygenase (PF13738) | 2.05 | 3.54 | 2.48 |
| **396511** | Oxidoreductases | Glucooligosaccharide oxidase (AA7) (**secreted**) | 18.94 | 17.20 | 5.40 |
| **186824** | Oxidoreductases | LPMOs (AA9, formerly GH61) (**secreted**) | 2.30 | 1.34 | 1.15 |
| **294705** | Oxidoreductases | LPMOs (AA9, formerly GH61) **(secreted**) | 13.65 | 9.58 | 12.54 |
| 694245 | Oxidoreductases | Monooxygenase, FAD-binding domain (**PF01494**) (**secreted**) | 3.79 | 1.60 | 0.92 |
| 315146 | Oxidoreductases | Peroxidase, Linoleate diol synthase (PGHS like) | 6.73 | 7.31 | 7.05 |
| 481621 | Signal transduction | GTPase-activator protein domain (PF00616) | 2.17 | 3.81 | 7.27 |
| 639980 | Signal transduction | Protein kinase domain (PF00069) | 3.42 | 0.77 | 0.78 |
| 252824 | Signal transduction | Protein kinase domain (PF00069) | 4.45 | 5.45 | 6.00 |
| 691907 | Signal transduction | Protein kinase domain (PF00069) | 2.52 | 1.48 | 0.68 |
| 706256 | Signal transduction | Serine/threonine protein kinase (putative) (**PF07714**) | 2.02 | 0.92 | 0.60 |
| 383113 | Cellular transport | ABC transporter protein family (PF00004,PF08740) | 3.56 | 1.83 | 0.98 |
| 250508 | Cellular transport | ABC transporter protein family (PF00005,PF00664) | 2.56 | 2.32 | 1.49 |
| 633182 | Cellular transport | ABC transporter protein family (PF00005,PF00664) | 2.41 | 33.39 | 22.79 |
| 688973 | Cellular transport | Citrate transporter domain (PF03600) | 2.70 | 2.66 | 1.64 |
| 650352 | Cellular transport | H+/nucleoside cotransporter (PF01773,PF07662) | 2.31 | 11.41 | 19.66 |
| 191443 | Cellular transport | H+-transporting ATPase (**PF00122**,**PF00690,PF00702**) | 4.08 | 0.36 | 0.13 |
| 482899 | Cellular transport | K+ potassium transporter (PF02705) | 3.94 | 3.20 | 0.96 |
| 250154 | Cellular transport | Major Facilitator Superfamily transporter (**PF07690**) | 2.71 | 1.54 | 0.91 |
| 598603 | Cellular transport | Major Facilitator Superfamily transporter (**PF07690**) | 2.46 | 8.51 | 7.47 |
| 482072 | Cellular transport | Major intrinsic protein family (PF00230) | 9.45 | 10.78 | 5.89 |
| *186573* ^e^ | Cellular transport | Sodium-potassium ATPase (**PF00122**,**PF00689**,**PF00690,PF00702**) | 4.33 | 2.20 | 0.70 |
| **659815** | Cellular transport | Sugar (and other) transporter (PF00083) | 2.35 | 1.88 | 1.74 |
| **677598** | Cellular transport | Sugar (and other) transporter (PF00083) | 13.57 | 27.20 | 14.83 |
| **702552** | Cellular transport | Hexose importer (LbMST1.3) (PF00083) | 2.61 | 5.42 | 5.04 |
| 186401 | Cellular transport | Sulfate permease | 2.42 | 1.42 | 0.64 |
| 192602 | Cytoskeleton  (Miscellaneous) | Profilin (PF00235) | 2.05 | 3.02 | 2.92 |
| 709998 | Protein turnover | Ubiquitin-activating enzyme, E1 | 2.17 | 0.73 | 0.86 |
| 676090 | Protein turnover | Ubiquitin-conjugating enzyme, E2 (**PF00179**) | 2.48 | 3.07 | 1.17 |
| 469445 | Protein folding | Cyclophilin (PF00160) | 2.73 | 0.65 | 0.59 |
| 443613 | Chaperone | HSP20 (PF00011) | 3.25 | 0.38 | 0.26 |
| 474704 | Chaperone | HSP20 (PF00011) | 2.06 | 0.47 | 0.23 |
| 675060 | Chaperone | HSP20 (PF00011) | 2.15 | 0.39 | 0.25 |
| 678280 | Chaperone | HSP20 (PF00011) | 3.02 | 0.59 | 0.37 |
| 706828 | Chaperone | HSP20 (PF00011) | 2.40 | 0.57 | 0.35 |
| 697312 | Transcription | Fungal Zn(2)-Cys(6) domain protein (**PF00172**) | 2.69 | 6.83 | 7.92 |
| 314000 | Transcription | Fungal specific transcription factor domain (PF04048) | 2.20 | 2.38 | 1.69 |
| 705705 | Transcription | TATA-binding protein interacting (TIP20) (PF08623) | 2.67 | 3.04 | 1.18 |
| 687436 | Translation | Translation initiation factor 5 | 4.54 | 5.73 | 3.73 |
| 294420 | RNA processing | Dicer endoribonuclease; RNase III family | 2.18 | 2.59 | 2.58 |
| 315790 | RNA processing | RNA-directed RNA polymerase (PF05183) | 2.38 | 2.80 | 3.52 |
| 313028 | Mating (Miscellaneous) | Pheromone protein domain (PF08015) | 2.49 | 1.16 | 1.30 |
| 192000 | Mating (Miscellaneous) | STE3-like pheromone receptor (PF02076) | 2.65 | 2.07 | 2.74 |
| 592787 | Poorly characterized | 2OG-Fe(II) oxygenase superfamily (PF03171) | 2.23 | 1.90 | 2.13 |
| 706230 | Poorly characterized | 2OG-Fe(II) oxygenase superfamily (PF03171) | 2.23 | 1.13 | 0.55 |
| 649610 | Poorly characterized | 50S ribosome-binding GTPase (PF01926) | 3.50 | 3.32 | 1.52 |
| 592437 | Poorly characterized | AAA domains (PF13476,PF13558) | 2.31 | 3.26 | 3.61 |
| 696517 | Poorly characterized | AAA domains (PPF13086,PF13087) | 3.85 | 1.70 | 2.54 |
| 300941 | Poorly characterized | BAG domain (PF02179) | 3.03 | 0.32 | 0.20 |
| 624778 | Poorly characterized | BTB/POZ domain (PF00651) | 2.22 | 1.79 | 2.13 |
| 668420 | Poorly characterized | BTB/POZ domain (PF00651) | 2.17 | 1.63 | 1.17 |
| 293242 | Poorly characterized | bZIP domain (PF00170) | 2.18 | 1.26 | 0.36 |
| 634481 | Poorly characterized | CRAL-TRIO domain (PF00650,PF03765) | 2.05 | 1.27 | 1.36 |
| 311783 | Poorly characterized | Dienelactone hydrolase family (PF01738) | 2.67 | 2.65 | 2.14 |
| 295833 | Poorly characterized | DUF2235 (**PF09994**) | 2.22 | 3.87 | 3.86 |
| 704758 | Poorly characterized | DUF2235 (**PF09994**) | 2.34 | 3.67 | 2.68 |
| 702586 | Poorly characterized | DUF2235 (**PF09994**) | 3.69 | 5.52 | 3.82 |
| 459272 | Poorly characterized | DUF3638,DUF3645 (PF12340,PF12359) | 2.63 | 2.13 | 1.63 |
| 449989 | Poorly characterized | DUF4243 (**PF14027**) | 2.94 | 5.24 | 5.88 |
| 311723 | Poorly characterized | Dynamin family protein (GTPase ) (**PF00350**,PF01031,PF02212) | 2.33 | 2.18 | 2.31 |
| 300342 | Poorly characterized | Exonuclease domain (PF00929) | 3.32 | 0.21 | 0.16 |
| 293762 | Poorly characterized | F-box-like domain (**PF12937**) **(secreted**) | 2.79 | 4.56 | 7.51 |
| 590848 | Poorly characterized | FGE-sulfatase domain (PF03781) | 2.25 | 3.00 | 2.08 |
| 707163 | Poorly characterized | FGE-sulfatase domain (PF03781) | 2.77 | 1.61 | 1.09 |
| 672925 | Poorly characterized | Helix-turn-helix domain (PF13560) | 4.08 | 15.17 | 14.19 |
| 458057 | Poorly characterized | HMG (high mobility group) box domain (PF00505) | 2.57 | 11.21 | 13.04 |
| 658142 | Poorly characterized | HMG (high mobility group) box domain (PF00505) | 3.46 | 8.75 | 13.01 |
| 311781 | Poorly characterized | Iron-containing alcohol dehydrogenase (PF00465) | 2.03 | 5.68 | 5.78 |
| 681029 | Poorly characterized | LCCL domain (PF03815) | 2.14 | 8.06 | 19.92 |
| 707489 | Poorly characterized | Mitochondrial chaperone BCS1 domain (PF08740) | 2.06 | 1.28 | 1.04 |
| 293824 | Poorly characterized | Peptidase inhibitor I48 domain (PF10467) | 4.09 | 5.31 | 3.90 |
| 326032 | Poorly characterized | Peptidase inhibitor I48 domain (PF10467) | 3.76 | 5.43 | 6.08 |
| 385833 | Poorly characterized | Peptidase inhibitor I48 domain (PF10467) | 8.05 | 20.80 | 10.31 |
| 602958 | Poorly characterized | Peptidase inhibitor I48 domain (PF10467) | 9.36 | 5.38 | 3.27 |
| 456319 | Poorly characterized | Phosphatidylinositol-4,5-bisphosphate 3-kinase (PF00454) and FAT (PF02259) domains | 2.35 | 3.83 | 8.00 |
| 579110 | Poorly characterized | PLAC8 domain (PF04749) | 2.18 | 2.23 | 2.70 |
| 656886 | Poorly characterized | SBDS domain (PF01172) | 3.93 | 4.17 | 3.88 |
| 655601 | Poorly characterized | SDR domain protein **(PF00106**) | 2.05 | 0.78 | 0.37 |
| 455673 | Poorly characterized | Secretory carrier membrane protein family (PF04144) | 2.30 | 1.16 | 0.79 |
| 688849 | Poorly characterized | SH3 domain (PF00018) | 2.85 | 2.16 | 1.33 |
| 192587 | Poorly characterized | Thaumatin like protein (**secreted**) | 2.56 | 0.80 | 0.42 |
| 439767 | Poorly characterized | Ubiquitin domain (PF00240), Zinc finger, C3HC4 type (PF13920) | 2.74 | 1.50 | 1.21 |
| 659443 | Poorly characterized | von Willebrand factor type A domain (PF13519) | 6.46 | 7.88 | 2.51 |
| 671119 | Poorly characterized | von Willebrand factor type A domain (PF13519) | 2.66 | 1.31 | 0.88 |

^*^ Proteins in bold letters are displayed in Fig. 3.

^†^ Underlined are *P. involutus* proteins that have a 1:1 orthologue identified in the *L. bicolor* genome.

^‡^ PFAM domains in bold letters represent 12 domains that were identified among the most highly up-regulated and expressed transcripts at the onset of SOM oxidation in both *P. involutus* and *L. bicolor*.

^§^ Predicted secretion signal.

^¶^ Protein id in italics indicates a 1:1 orthologue that was found among the up-regulated and expressed transcripts at the onset of SOM oxidation in both *L. bicolor*  and *P. involutus* (cf. Table S7).

**Table S9.** Genes up-regulated during ammonium limitation in *Saccharomyces cerevisiae* (Sc) that display sequence similarities to genes in *Paxillus involutus* (PAI) and *Laccaria bicolor* (LAB). Sc gene descriptions are according to the Saccharomyces Genome Database (SGD; www.yeastgenome.org).

| **Sc gene** |  | **Description** | **PAI**  **Prot id** | **t_2_ vs t_1_^*^** | **LAB**  **Prot id** | **t_2_ vs t_1_^*^** |
| --- | --- | --- | --- | --- | --- | --- |
| *CPS1* | *YJL172W* | Vacuolar carboxypeptidase S | 66863 |  | 182303 |  |
| *GLN1* | *YPR035W* | Glutamine synthetase (GS); | 166353 |  | 183088 |  |
| *GLT1* | *YDL171C* | NAD(+)-dependent glutamate synthase (GOGAT) | 73255 |  | 183838 | 0.61 |
| *DAL5* | *YJR152W* | Allantoate permease | 167364 | 0.73 | 187852 |  |
| *CAN1* | *YEL063C* | Plasma membrane arginine permease | 168793 | **1.15** | 188015 | 0.83 |
| *PDC5* | *YLR134W* | Pyruvate decarboxylase | 175618 |  | 188370 |  |
| *APE1* | *YKL103C* | Vacuolar aminopeptidase (peptidase family M18) | 67584 |  | 188577 |  |
| *PUT2* | *YHR037W* | Delta-1-pyrroline-5-carboxylate dehydrogenase | 68289 |  | 188701 | **1.56** |
| *-* | *YBR139W* | Putative serine type carboxypeptidase | 169745 | 0.49 | 189535 | 0.74 |
| *ENA1* | *YDR040C* | P-type ATPase sodium pump | 168107 |  | 189612 |  |
| *MEP2* | *YNL142W* | Ammonium permease | 15976 |  | 190906 |  |
| *VBA1* | *YMR088C* | Permease of basic amino acids (vacuolar membrane) | 160604 |  | 245814 | 0.75 |
| *DAL4* | *YIR028W* | Allantoin permease | 114363 | 1.41 | 248955 | 0.41 |
| *UGA4* | *YDL210W* | GABA permease (vacuolar membrane) | 114291 | 0.96 | 254728 |  |
| *ALD2* | *YMR170C* | Cytoplasmic aldehyde dehydrogenase | 76843 |  | 256254 |  |
| *GDH1 (GDH3)* | *YOR375C* | NADP(+)-dependent glutamate dehydrogenase | ND |  | 292653 |  |
| *CPA1* | *YOR303W* | Carbamoyl phosphate synthetase | 166769 |  | 294748 |  |
| *ARG1* | *YOL058W* | Arginosuccinate synthetase | 160149 |  | 294826 |  |
| *GAP1* | *YKR039W* | General amino acid permease | 66349 |  | 296817 | **2.45** |
| *PUT4* | *YOR348C* | Proline permease | 165046 |  | 300838 |  |
| *MEP1 (MEP3)* | *YGR121C* | Ammonium permease | 164520 | 1.89 | 300932 |  |
| *OPT2* | *YPR194C* | Oligopeptide transporter (localized to peroxisomes) | 166564 | 0.51 | 302225 |  |
| *GDH2* | *YDL215C* | NAD(+)-dependent glutamate dehydrogenase | 9798 |  | 309733 |  |
| *DAL7, MLS1* | *YIR031C* | Malate synthase | 128854 |  | 314107 |  |
| *DUR3* | *YHL016C* | Plasma membrane transporter for both urea and polyamines | 172505 | **1.25** | 315056 |  |
| *IDP1* | *YDL066W* | Isocitrate dehydrogenase | 168511 |  | 317084 |  |
| *PRB1* | *YEL060C* | Vacuolar proteinase B | 18477 |  | 396035 |  |
| *DUR1,2* | *YBR208C* | Urea amidolyase | 106152 |  | 399837 | 1.13 |
| *-* | *YDR090C* | Putative protein of unknown function | ND |  | 608302 |  |
| *AMD2* | *YDR242W* | Putative amidase | 11929 |  | 622159 |  |
| *AVT4* | *YNL101W* | Vacuolar transporter | 170470 |  | 629310 |  |
| *UGA2* | *YBR006W* | Succinate semialdehyde dehydrogenase | 96663 |  | 656822 |  |
| *DAL2* | *YIR029W* | Allantoicase | 166992 | 0.84 | 696602 |  |
| *RPL7B* | *YPL198W* | Ribosomal 60S subunit protein L7B | 20437 |  | 702133 |  |
| *YGK3* | *YOL128C* | Protein kinase related to mammalian GSK-3 glycogen synthase kinases | 168023 |  | 702204 |  |
| *LAP3* | *YNL239W* | Cysteine aminopeptidase (bleomycin hydrolase) | 69875 | **2.28** | 635298 | **4.81** |
| *DAL1* | *YIR027C* | Allantoinase | 167262 |  | 709233 |  |
| *ECM38* | *YLR299W* | Gamma-glutamyltranspeptidase | 128053 | 0.40 | 709265 |  |
| *DCG1* | *YIR030C* | Protein of unknown function | 65065 |  | ND |  |
| *BSC6* | *YOL137W* | Protein of unknown function | 125972 |  | ND |  |
| *SDS23* | *YGL056C* | Protein involved in cell separation | 162950 | 0.70 | ND |  |

^*^Log_2_-fold changes in gene expression, t_2_ versus t_1_. Only fold changes that were significantly up-regulated are shown (*p*_adj_≤0.01, *n*=3). Fold changes in bold letters indicate that the genes were found among the most highly up-regulated and expressed genes at the onset of SOM oxidation in *P. involutus* (Table S7) and *L. bicolor* (Table S8), respectively.

**Table S10.** Identification of homologs displayed in Fig. 3. PAI, *Paxillus involutus*; LAB, *Laccaria bicolor*.

| **Gene description** | **PAI**  **Prot id** | **LAB**  **Prot id** | **Criteria for identifying homologs** |
| --- | --- | --- | --- |
| Nitrate transporter | 76416 | 254042 | 1:1 orthologs |
| Oligopeptide transporter | 164294 | 190652 | 1:1 orthologs |
| Cysteine aminopeptidase (*LAP3*) | 69875 | 635298 | *P. involutus* has one gene copy. *L. bicolor* has two copies, displayed is the copy that is highest upregulated at t_2_. |
| A01 Peptidase | 105760 | 250557 | 1:1 orthologs |
| Ammonium permease | 15976 | 188643 | 1:1 orthologs |
| 3-deoxy-7-phosphoheptulonate synthase | 167454 | 649395 | 1:1 orthologs |
| Urea and polyamine transporter (*Dur3*) | 172505 | 315056 | 1:1 orthologs |
| Amino acid permease | 168793 | 300838 | Phylogeny, Fig. S7 |
| 1-pyrroline-5-carboxylate dehydrogenase (*PUT2*) | 68289 | 188701 | 1:1 orthologs |
| Glutamate dehydrogenase | 9798 | 182694 | 1:1 orthologs |
| Oligopeptide transporter | 151971 | 242631 | 1:1 orthologs |
| A01 Peptidase | 167076 | 144077 | Phylogeny in Shah et al. 2016 (New Phytol 209:1705). Two close homologs in *L. bicolor*, shown is the fold values of the model that is highest upregulated at t_2._ |
| Amino acid permease | No | 296817 | Phylogeny, Fig. S7 |
| Pectin methylesterase (CE8) | No | 245379 | According to annotations in Kohler et al 2015  (Nat Genet 47:410). |
| Amylase (GH13_1) | 10103 | 247310 | 1:1 orthologs |
| Lysozyme (GH24) | No | 239749 | According to annotations in Kohler et al 2015  (Nat Genet 47:410). |
| A-A trehalase GH37 | 33997 | 245054 | 1:1 orthologs |
| AA5_1 (CRO-1) | No | 313935 | According to annotations in Kohler et al 2015  (Nat Genet 47:410). |
| Glucooligosaccharide oxidase (AA7) | No | 396511 | According to annotations in Kohler et al 2015  (Nat Genet 47:410). |
| LPMO (AA9) | 168805 | 186824 | 1:1 orthologs |
| LPMO (AA9) | 133034 | 294705 | 1:1 orthologs |
| Lysozyme/Chitinase (GH23) | No | 313808 | According to annotations Kohler et al 2015  (Nat Genet 47:410). |
| Chitin deacetylase (CE4) | 161071 | 253895 | 1:1 orthologs |
| Chitin deacetylase (CE4) | No | 307596 | No sequence in *P. involutus* with sequence similarity (cut-off: <1.00E-20, BLASTP) |
| Chitin deacetylase (CE4) | No | 293318 | No sequence in *P. involutus* with sequence similarity (cut-off: <1.00E-20, BLASTP) |
| Chitin deacetylase (CE4) | No | 308312 | No sequence in *P. involutus* with sequence similarity (cut-off: <1.00E-20, BLASTP) |
| Sugar (and other) transporter | 163069 | 677598 | 1:1 orthologs |
| Sugar (and other) transporter | 130952 | 659815 | 1:1 orthologs |
| Hexose importer (LbMST1.3) | 169976 | 702552 | Two possible homologs in *P. involutus*, shown is the model that is highest upregulated at t_2_. |

**Table S11.** Response types significance testing. The 15 discovered response types were tested for both enrichment and depletion for genes in each of the two main annotation categories, metabolism and SOM interaction, for both fungi using a permutation test (see Supporting Information). The column `#genes in response type by annot.` shows the number of unique genes in the response type which are directly clustered within the main annotation category i.e. disregarding genes clustered at the root annotation level. `Expected by chance` is the number of genes expected under the null hypothesis of memoryless conditions. All the obtained *p-*values were corrected together for multiple testing to control the family-wise error rate (column *p*_adj_). PAI, *Paxillus involutus*; LAB, *Laccaria bicolor*.

| **Species** | **Response type** | **Main annotation**  **category** | **Testing for** | **Significant 5%** | **#genes in response type by annot.** | **Expected by chance** | ***p*-value** | ***p*_adj_** |
| --- | --- | --- | --- | --- | --- | --- | --- | --- |
| PAI | r_1_ | KEGG Metabolism | enrichment | no | 0 | 1 | 1 | 1 |
| PAI | r_1_ | SOM interactions | enrichment | no | 4 | 1 | 0.007125703833832661 | 0.5914334182081108 |
| PAI | r_1_ | KEGG Metabolism | depletion | no | 0 | 1 | 0.41729603878790056 | 1 |
| PAI | r_1_ | SOM interactions | depletion | no | 4 | 1 | 0.998974725957329 | 1 |
| LAB | r_1_ | KEGG Metabolism | enrichment | no | 4 | 2 | 0.1422913042841638 | 1 |
| LAB | r_1_ | SOM interactions | enrichment | no | 10 | 5 | 0.028220989552960637 | 1 |
| LAB | r_1_ | KEGG Metabolism | depletion | no | 4 | 2 | 0.9478452521089685 | 1 |
| LAB | r_1_ | SOM interactions | depletion | no | 10 | 5 | 0.9881827737533291 | 1 |
| PAI | r_2_ | KEGG Metabolism | enrichment | no | 6 | 4 | 0.22009859266131382 | 1 |
| PAI | r_2_ | SOM interactions | enrichment | no | 3 | 1 | 0.03985837707382089 | 1 |
| PAI | r_2_ | KEGG Metabolism | depletion | no | 6 | 4 | 0.886454571809395 | 1 |
| PAI | r_2_ | SOM interactions | depletion | no | 3 | 1 | 0.9929135851228119 | 1 |
| LAB | r_2_ | KEGG Metabolism | enrichment | enriched | 29 | 7 | 5.373385193971296e-10 | 5.642054453669861e-8 |
| LAB | r_2_ | SOM interactions | enrichment | enriched | 15 | 4 | 8.126905841255394e-6 | 7.801829607605177e-4 |
| LAB | r_2_ | KEGG Metabolism | depletion | no | 29 | 7 | 0.9999999998780269 | 1 |
| LAB | r_2_ | SOM interactions | depletion | no | 15 | 4 | 0.9999981794174633 | 1 |
| PAI | r_3_ | KEGG Metabolism | enrichment | no | 0 | 27 | 1 | 1 |
| PAI | r_3_ | SOM interactions | enrichment | no | 0 | 12 | 1 | 1 |
| PAI | r_3_ | KEGG Metabolism | depletion | depleted | 0 | 27 | 6.693487562092438e-13 | 7.228966567059833e-11 |
| PAI | r_3_ | SOM interactions | depletion | depleted | 0 | 12 | 3.781959572944938e-6 | 3.781959572944938e-4 |
| LAB | r_3_ | KEGG Metabolism | enrichment | no | 0 | 36 | 1 | 1 |
| LAB | r_3_ | SOM interactions | enrichment | no | 0 | 25 | 1 | 1 |
| LAB | r_3_ | KEGG Metabolism | depletion | depleted | 0 | 36 | 5.816843648554603e-17 | 6.514864886381155e-15 |
| LAB | r_3_ | SOM interactions | depletion | depleted | 0 | 25 | 9.724093957071962e-12 | 1.0404780534067e-9 |
| PAI | r_4_ | KEGG Metabolism | enrichment | no | 3 | 6 | 0.9480132204451287 | 1 |
| PAI | r_4_ | SOM interactions | enrichment | no | 0 | 15 | 1 | 1 |
| PAI | r_4_ | KEGG Metabolism | depletion | no | 3 | 6 | 0.13157126120379248 | 1 |
| PAI | r_4_ | SOM interactions | depletion | depleted | 0 | 15 | 2.6005257154820153e-7 | 2.6525362297916556e-5 |
| LAB | r_4_ | KEGG Metabolism | enrichment | no | 0 | 8 | 1 | 1 |
| LAB | r_4_ | SOM interactions | enrichment | no | 0 | 20 | 1 | 1 |
| LAB | r_4_ | KEGG Metabolism | depletion | depleted | 0 | 8 | 3.2789285419449344e-4 | 0.030166142585893396 |
| LAB | r_4_ | SOM interactions | depletion | depleted | 0 | 20 | 1.4069157048105549e-9 | 1.4631923330029772e-7 |
| PAI | r_5_ | KEGG Metabolism | enrichment | no | 0 | 24 | 1 | 1 |
| PAI | r_5_ | SOM interactions | enrichment | no | 9 | 12 | 0.8151823524249573 | 1 |
| PAI | r_5_ | KEGG Metabolism | depletion | depleted | 0 | 24 | 2.4314698584608976e-11 | 2.5773580499685515e-9 |
| PAI | r_5_ | SOM interactions | depletion | no | 9 | 12 | 0.28348170087885294 | 1 |
| LAB | r_5_ | KEGG Metabolism | enrichment | no | 13 | 29 | 0.999801733793312 | 1 |
| LAB | r_5_ | SOM interactions | enrichment | no | 24 | 21 | 0.3090193445599406 | 1 |
| LAB | r_5_ | KEGG Metabolism | depletion | depleted | 13 | 29 | 4.8730942383031915e-4 | 0.04434515756855904 |
| LAB | r_5_ | SOM interactions | depletion | no | 24 | 21 | 0.7618280447571817 | 1 |
| PAI | r_6_ | KEGG Metabolism | enrichment | enriched | 8 | 1 | 5.07344274927894e-6 | 4.971973894293361e-4 |
| PAI | r_6_ | SOM interactions | enrichment | no | 6 | 3 | 0.11973575825510974 | 1 |
| PAI | r_6_ | KEGG Metabolism | depletion | no | 8 | 1 | 0.9999995005806531 | 1 |
| PAI | r_6_ | SOM interactions | depletion | no | 6 | 3 | 0.9480041269330145 | 1 |
| LAB | r_6_ | KEGG Metabolism | enrichment | no | 0 | 3 | 1 | 1 |
| LAB | r_6_ | SOM interactions | enrichment | no | 0 | 7 | 1 | 1 |
| LAB | r_6_ | KEGG Metabolism | depletion | no | 0 | 3 | 0.058346358383302654 | 1 |
| LAB | r_6_ | SOM interactions | depletion | no | 0 | 7 | 9.867566959510597e-4 | 0.08683458924369325 |
| PAI | r_7_ | KEGG Metabolism | enrichment | no | 0 | 0 | 1 | 1 |
| PAI | r_7_ | SOM interactions | enrichment | enriched | 24 | 3 | 2.0068308486816792e-14 | 2.2275822420366638e-12 |
| PAI | r_7_ | KEGG Metabolism | depletion | no | 0 | 0 | 0.7819812431668974 | 1 |
| PAI | r_7_ | SOM interactions | depletion | no | 24 | 3 | 0.9999999999999978 | 1 |
| LAB | r_7_ | KEGG Metabolism | enrichment | enriched | 25 | 4 | 1.4467757491473237e-13 | 1.576985566570583e-11 |
| LAB | r_7_ | SOM interactions | enrichment | enriched | 53 | 8 | 2.413333111688129e-26 | 2.751199747324467e-24 |
| LAB | r_7_ | KEGG Metabolism | depletion | no | 25 | 4 | 0.9999999999999807 | 1 |
| LAB | r_7_ | SOM interactions | depletion | no | 53 | 8 | 1 | 1 |
| PAI | r_8_ | KEGG Metabolism | enrichment | no | 14 | 12 | 0.31248920341984365 | 1 |
| PAI | r_8_ | SOM interactions | enrichment | no | 0 | 5 | 1 | 1 |
| PAI | r_8_ | KEGG Metabolism | depletion | no | 14 | 12 | 0.7787384667026171 | 1 |
| PAI | r_8_ | SOM interactions | depletion | no | 0 | 5 | 0.004996459323368264 | 0.42469904248630247 |
| LAB | r_8_ | KEGG Metabolism | enrichment | no | 0 | 15 | 1 | 1 |
| LAB | r_8_ | SOM interactions | enrichment | no | 0 | 12 | 1 | 1 |
| LAB | r_8_ | KEGG Metabolism | depletion | depleted | 0 | 15 | 2.45925905461603e-7 | 2.533036826254511e-5 |
| LAB | r_8_ | SOM interactions | depletion | depleted | 0 | 12 | 6.377740349042393e-6 | 6.186408138571121e-4 |
| PAI | r_9_ | KEGG Metabolism | enrichment | no | 0 | 1 | 1 | 1 |
| PAI | r_9_ | SOM interactions | enrichment | enriched | 6 | 1 | 6.748031949764896e-5 | 0.006275669713281353 |
| PAI | r_9_ | KEGG Metabolism | depletion | no | 0 | 1 | 0.558385671760614 | 1 |
| PAI | r_9_ | SOM interactions | depletion | no | 6 | 1 | 0.999993755705491 | 1 |
| LAB | r_9_ | KEGG Metabolism | enrichment | enriched | 27 | 2 | 5.038159967240189e-23 | 5.693120762981414e-21 |
| LAB | r_9_ | SOM interactions | enrichment | enriched | 92 | 5 | 1.910843038362526e-88 | 2.2165779245005304e-86 |
| LAB | r_9_ | KEGG Metabolism | depletion | no | 27 | 2 | 1 | 1 |
| LAB | r_9_ | SOM interactions | depletion | no | 92 | 5 | 1 | 1 |
| PAI | r_10_ | KEGG Metabolism | enrichment | enriched | 248 | 11 | 2.0408068396267148e-286 | 2.4285601391557907e-284 |
| PAI | r_10_ | SOM interactions | enrichment | enriched | 107 | 5 | 8.988677386211327e-110 | 1.0516752541867252e-107 |
| PAI | r_10_ | KEGG Metabolism | depletion | no | 248 | 11 | 1 | 1 |
| PAI | r_10_ | SOM interactions | depletion | no | 107 | 5 | 1 | 1 |
| LAB | r_10_ | KEGG Metabolism | enrichment | enriched | 314 | 14 | 0 | 0 |
| LAB | r_10_ | SOM interactions | enrichment | enriched | 173 | 8 | 1.4915251756312908e-174 | 1.759999707244923e-172 |
| LAB | r_10_ | KEGG Metabolism | depletion | no | 314 | 14 | 1 | 1 |
| LAB | r_10_ | SOM interactions | depletion | no | 173 | 8 | 1 | 1 |
| PAI | r_11_ | KEGG Metabolism | enrichment | no | 0 | 6 | 1 | 1 |
| PAI | r_11_ | SOM interactions | enrichment | no | 3 | 9 | 0.9934712380318691 | 1 |
| PAI | r_11_ | KEGG Metabolism | depletion | no | 0 | 6 | 0.0036040712897859348 | 0.3099501309215904 |
| PAI | r_11_ | SOM interactions | depletion | no | 3 | 9 | 0.022370234429340965 | 1 |
| LAB | r_11_ | KEGG Metabolism | enrichment | no | 0 | 0 | 1 | 1 |
| LAB | r_11_ | SOM interactions | enrichment | enriched | 9 | 2 | 2.681942035511703e-5 | 0.0025478449337361176 |
| LAB | r_11_ | KEGG Metabolism | depletion | no | 0 | 0 | 0.9755528865698316 | 1 |
| LAB | r_11_ | SOM interactions | depletion | no | 9 | 2 | 0.9999960945159225 | 1 |
| PAI | r_12_ | KEGG Metabolism | enrichment | no | 37 | 24 | 0.0076710688472891924 | 0.6290276454777137 |
| PAI | r_12_ | SOM interactions | enrichment | enriched | 93 | 17 | 1.047322837075984e-43 | 1.2044212626373815e-41 |
| PAI | r_12_ | KEGG Metabolism | depletion | no | 37 | 24 | 0.9954570710852759 | 1 |
| PAI | r_12_ | SOM interactions | depletion | no | 93 | 17 | 1 | 1 |
| LAB | r_12_ | KEGG Metabolism | enrichment | no | 16 | 29 | 0.9968169012240014 | 1 |
| LAB | r_12_ | SOM interactions | enrichment | enriched | 43 | 20 | 2.8410425974207478e-6 | 2.8694530233949555e-4 |
| LAB | r_12_ | KEGG Metabolism | depletion | no | 16 | 29 | 0.006252376934351249 | 0.5251996624855049 |
| LAB | r_12_ | SOM interactions | depletion | no | 43 | 20 | 0.9999988012742438 | 1 |
| PAI | r_13_ | KEGG Metabolism | enrichment | enriched | 20 | 2 | 5.6689250210911384e-14 | 6.235817523200252e-12 |
| PAI | r_13_ | SOM interactions | enrichment | enriched | 16 | 4 | 4.325085438926747e-6 | 4.281834584537479e-4 |
| PAI | r_13_ | KEGG Metabolism | depletion | no | 20 | 2 | 0.9999999999999949 | 1 |
| PAI | r_13_ | SOM interactions | depletion | no | 16 | 4 | 0.9999990330412012 | 1 |
| LAB | r_13_ | KEGG Metabolism | enrichment | enriched | 16 | 5 | 4.5217750670805326e-5 | 0.0042504685630557005 |
| LAB | r_13_ | SOM interactions | enrichment | no | 17 | 11 | 0.0696439415528858 | 1 |
| LAB | r_13_ | KEGG Metabolism | depletion | no | 16 | 5 | 0.9999876222873632 | 1 |
| LAB | r_13_ | SOM interactions | depletion | no | 17 | 11 | 0.959168848542131 | 1 |
| PAI | r_14_ | KEGG Metabolism | enrichment | no | 9 | 4 | 0.03579183449770765 | 1 |
| PAI | r_14_ | SOM interactions | enrichment | no | 0 | 1 | 1 | 1 |
| PAI | r_14_ | KEGG Metabolism | depletion | no | 9 | 4 | 0.9852514648836612 | 1 |
| PAI | r_14_ | SOM interactions | depletion | no | 0 | 1 | 0.4733913479232288 | 1 |
| LAB | r_14_ | KEGG Metabolism | enrichment | no | 0 | 7 | 1 | 1 |
| LAB | r_14_ | SOM interactions | enrichment | no | 0 | 4 | 1 | 1 |
| LAB | r_14_ | KEGG Metabolism | depletion | no | 0 | 7 | 6.72724330537771e-4 | 0.05987246541786161 |
| LAB | r_14_ | SOM interactions | depletion | no | 0 | 4 | 0.023297203185207113 | 1 |
| PAI | r_15_ | KEGG Metabolism | enrichment | no | 6 | 1 | 5.655695206039917e-4 | 0.050901256854359256 |
| PAI | r_15_ | SOM interactions | enrichment | no | 4 | 1 | 0.030872935277492332 | 1 |
| PAI | r_15_ | KEGG Metabolism | depletion | no | 6 | 1 | 0.9999222137854645 | 1 |
| PAI | r_15_ | SOM interactions | depletion | no | 4 | 1 | 0.9931618652175196 | 1 |
| LAB | r_15_ | KEGG Metabolism | enrichment | no | 0 | 3 | 1 | 1 |
| LAB | r_15_ | SOM interactions | enrichment | no | 0 | 6 | 1 | 1 |
| LAB | r_15_ | KEGG Metabolism | depletion | no | 0 | 3 | 0.07494169908519145 | 1 |
| LAB | r_15_ | SOM interactions | depletion | no | 0 | 6 | 0.002023317711429996 | 0.17602864089440964 |

**References**

1. Rineau F, Roth D, Shah F, Smits M, Johansson T, Canbäck B *et al*. The ectomycorrhizal fungus *Paxillus involutus* converts organic matter in plant litter using a trimmed brown-rot mechanism involving Fenton chemistry. *Environ Microbiol* 2012; **14**: 1477-1487.

2. Shah F, Rineau F, Canbäck B, Johansson T, Tunlid A. The molecular components of the extracellular protein-degradation pathways of the ectomycorrhizal fungus *Paxillus involutus*. *New Phytol* 2013; **200**: 875-887.

3. Davidson EA, Galloway LF, Strand, MK. Assessing available carbon: comparison of techniques across selected forest soils. *Commun Soil Sci Plant Anal* 1987; **18**: 45-65.

4. Buurman P, Jongman AG, Nierop KGJ. Comparison of Michigan and Dutch podzolized soils: organic matter characterization by micromorphology and pyrolysis-GC/MS. *Soil Sci Soc Am J* 2008; **72**: 1344-1356.

5. Webb SM. SIXpack: a graphical user interface for XAS analysis using IFEFFIT. *Phys Scr* 2005; **T115**: 1011-1014.

6. Wilke M, Farges F, Petit P-E, Brown Jr GE, Martin F. Oxidation state and coordination of Fe in minerals: An Fe K-XANES spectroscopic study. *Am Mineral* 2001; **86**: 714-730.

7. Wojdyr M. Fityk: a general-purpose peak fitting program. *J Appl Cryst* 2010; **43**:1126-1128.

8. Leinweber P, Kruse J, Walley FL, Gillespie A, Eckhardt KU, Blyth RI *et al.* Nitrogen K-edge XANES - an overview of reference compounds used to identify unknown organic nitrogen in environmental samples. *J Synchrotron Radiat* 2007; **14**: 500-511.

9. Jaumot J, Gargallo R, de Juan A, Tauler RA. Graphical user friendly interface for MCR-ALS: A new tool for multivariate curve resolution in MATLAB. *Chemom Intell Lab Syst* 2005; **76**: 101-110.

10. Felten J, Hall H, Jaumot J, Tauler R, de Juan A, Gorzsá A. Vibrational spectroscopic image analysis of biological material using multivariate curve resolution-alternating least squares (MCR-ALS). *Nat Protoc* 2015; **10**: 217-240.

11. Andrews S. *FastQC A Quality Control tool for High Throughput Sequence Data*. <http://www.bioinformatics.babraham.ac.uk/projects/fastqc/>, 2010.

12. Schmieder R, Lim YW, Edwards R. Identification and removal of ribosomal RNA sequences from metatranscriptomes. *Bioinformatics* 2012; **28**: 433-435.

13. Grigoriev IV, Nikitin R, Haridas S, Kuo A, Ohm R, Otillar R *et al*. MycoCosm portal: gearing up for 1000 fungal genomes. *Nucleic Acids Res* 2014; **42**: D699-D704.

14. Kim D, Pertea G, Trapnell C, Pimentel H, Kelley R, Salzberg SL. TopHat2: accurate alignment of transcriptomes in the presence of insertions, deletions and gene fusions. *Genome Biol* 2013; **14**(4): R36.

15. Langmead B, Salzberg SL. Fast gapped-read alignment with Bowtie 2. *Nat Methods* 2012; **9**: 357-359.

16. Li H, Handsaker B, Wysoker A, Fennell T, Ruan J, Homer N *et al.* The Sequence Alignment/Map format and SAMtools. *Bioinformatics* 2009; **25**: 2078-2079.

17. Anders S, Pyl PT, Huber W. HTSeq - a Python framework to work with high-throughput sequencing data. *Bioinformatics* 2015; **31**: 166-169.

18. Risso D, Schwartz K, Sherlock G, Dudoit S. GC-content normalization for RNA-Seq data. *BMC Bioinformatics* 2011; **12**: 480.

19. Love MI, Huber W, Anders S. Moderated estimation of fold change and dispersion for RNA-seq data with DESeq2. *Genome Biol* 2014; **15**(12): 550.

20. Young MD, Wakefield MJ, Smyth GK, Oshlack A. Gene ontology analysis for RNA-seq: accounting for selection bias. *Genome Biol* 2010; **11**(2): R14.

21. Benjamini Y, Hochberg Y. Controlling the false discovery rate: a practical and powerful approach to multiple testing. *JR Stat Soc Series B Methods* 1995; **57**: 289-300.

22. R Development Core Team. *R: A language and environment for statistical computing*. R Foundation for Statistical Computing, Vienna, Austria, 2016.

23. Sanavia T, Finotello F, Di CB. FunPat: function-based pattern analysis on RNA-seq time series data. *BMC Genomics* 2015; **16**: S2.

24. Ernst J, Nau GJ, Bar-Joseph Z. Clustering short time series gene expression data. *Bioinformatics* 2005; **21** Suppl 1:i159-i168.

25. Holm S. A simple sequentially rejective multiple test procedure. *Scandinavian Journal of Statistics* 1979; **6**: 65-70.

26. Rawlings ND, Barrett AJ, Finn R. Twenty years of the MEROPS database of proteolytic enzymes, their substrates and inhibitors. *Nucleic Acids Res* 2016; **44**: D343-D350.

27. Shah F, Nicolás C, Bentzer J, Ellström M, Smits M, Rineau F *et al*. Ectomycorrhizal fungi decompose soil organic matter using oxidative mechanisms adapted from saprotrophic ancestors. *New Phytol* 2016; **209**: 1705-1719.

28. Lucic E, Fourrey C, Kohler A, Martin F, Chalot M, Brun-Jacob A. A gene repertoire for nitrogen transporters in *Laccaria bicolor*. *New Phytol* 2008; **180**:343-364.

29. Kanehisa M, Sato Y, Morishima K. BlastKOALA and GhostKOALA: KEGG tools for functional characterization of genome and metagenome sequences. *J Mol Biol* 2016; **428**: 726-731.

30. Weber T, Blin K, Duddela S, Krug D, Kim HU, Bruccoleri R *et al*. antiSMASH 3.0 - a comprehensive resource for the genome mining of biosynthetic gene clusters. *Nucleic Acids Res* 2015; **43**: W237-W243..

31. Gattiker A, Gasteiger E, Bairoch A. ScanProsite: a reference implementation of a PROSITE scanning tool. *Appl Bioinformatics* 2002; **1**:107-108.

32. Cortazar AR, Aransay AM, Alfaro M, Oguiza JA, Lavín JL.SECRETOOL: integrated secretome analysis tool for fungi. *Amino Acids* 2014; **46**: 471-473.

33. Bendtsen JD, Nielsen H, von Heijne G, Brunak S. Improved prediction of signal peptides: SignalP 3.0. *J Mol Biol* 2004; **340**: 783-795.

34. Bendtsen JD, Jensen LJ, Blom N, Von Heijne G, Brunak S. Feature-based prediction of non-classical and leaderless protein secretion. *Protein Eng Des Sel* 2004; **17**: 349-356.

35. Petersen TN, Brunak S, von Heijne G, Nielsen H. SignalP 4.0: discriminating signal peptides from transmembrane regions. *Nat Methods* 2011; **8**: 785-786.

36. Emanuelsson O, Nielsen H, Brunak S, von Heijne G. Predicting subcellular localization of proteins based on their N-terminal amino acid sequence. *J Mol Biol* 2000; **300**: 1005-1016.

37. Krogh A, Larsson B, von Heijne G, Sonnhammer EL. Predicting transmembrane protein topology with a hidden Markov model: application to complete genomes. *J Mol Biol* 2001; **305**: 567-580.

38. Horton P, Park KJ, Obayashi T, Fujita N, Harada H, Adams-Collier CJ *et al.* WoLF PSORT: protein localization predictor. *Nucleic Acids Res* 2007; **35**: W585-W587.

39. Kall L, Krogh A, Sonnhammer EL. Advantages of combined transmembrane topology and signal peptide prediction--the Phobius web server. *Nucleic Acids Res* 2007; **35**: W429-W432.

40. Kall L, Krogh A, Sonnhammer EL. A combined transmembrane topology and signal peptide prediction method. *J Mol Biol* 2004; **338**: 1027-1036.

41. Boer VM, de Winde JH, Pronk JT, Piper MD. The genome-wide transcriptional responses of *Saccharomyces cerevisiae* grown on glucose in aerobic chemostat cultures limited for carbon, nitrogen, phosphorus, or sulfur. *J Biol Chem* 2003; **278**: 3265-3274.

42. Boer VM, Tai SL, Vuralhan Z, Arifin Y, Walsh MC, Piper MD *et al*. Transcriptional responses of *Saccharomyces cerevisiae* to preferred and nonpreferred nitrogen sources in glucose-limited chemostat cultures. *FEMS Yeast Res* 2007; **7**: 604-620.

43. Lechner M, Findeiß S, Steiner L, Marz M, Stadler PF, Prohaska SJ. Proteinortho: detection of (co-)orthologs in large-scale analysis. *BMC Bioinformatics* 2011; **12**:124.

44. Katoh K, Standley DM. MAFFT multiple sequence alignment software version 7: improvements in performance and usability. *Mol Biol Evol* 2013; **30**: 772-780.

45. Waterhouse AM, Procter JB, Martin DM, Clamp M, Barton GJ. Jalview Version 2--a multiple sequence alignment editor and analysis workbench. *Bioinformatics* 2009; **25**: 1189-1191.

46. Stamatakis A. RAxML version 8: a tool for phylogenetic analysis and post-analysis of large phylogenies. *Bioinformatics* 2014; **30**: 1312-1313.

47. Miller MA, Pfieffer W, Schwartz T. Creating the CIPRES Science Gateway for inference of large phylogenetic trees. In: *Proceedings of the Gateway Computing Environments Workshop (GCE)*; New Orleans, LA, USA, 2010, pp. 1-8.
